# Author Correction: Engineering self-organized criticality in living cells

**DOI:** 10.1038/s41467-021-25603-6

**Published:** 2021-09-23

**Authors:** Blai Vidiella, Antoni Guillamon, Josep Sardanyés, Victor Maull, Jordi Pla, Nuria Conde, Ricard Solé

**Affiliations:** 1 ICREA-Complex Systems Lab, Universitat Pompeu Fabra, Barcelona, Spain; 2 Institut de Biologia Evolutiva (CSIC-UPF), Barcelona, Spain; 3 Departament de Matemàtiques, EPSEB, Universitat Politècnica de Catalunya, Barcelona, Spain; 4 Institut de Matemàtiques de la UPC-BarcelonaTech (IMTech), Universitat Politècnica de Catalunya, Barcelona, Spain; 5 Centre de Recerca Matemàtica, Edifici C, Campus de Bellaterra, Barcelona, Spain; 6 Santa Fe Institute, Santa Fe NM, USA

**Keywords:** Criticality, Genetic circuit engineering

Correction to: *Nature Communications* 10.1038/s41467-021-24695-4, published online 20 July 2021.

The original version of this Article omitted the following from the Acknowledgements:

AEI-PID2019-111680GB-I00/AEI/10.13039/501100011033

This has now been corrected in both the PDF and HTML versions of the Article.

