# Supplementary material for: Engineering self-organized criticality in living cells
Source: Nat Commun. 2021 Jul 20;12:4415. doi: 10.1038/s41467-021-24695-4 (PMC8292319; doi:10.1038/s41467-021-24695-4)
Supplement: Supplementary file 1 — Supplementary Info [file 41467_2021_24695_MOESM1_ESM.pdf]

Engineering self-organized criticality in living cells

Supplementary material

Blai Vidiella,[1,2] Antoni Guillamon,[3,4,5] Josep Sardanyés,[5] Victor Maull,[1,2] Jordi Pla,[1,2] Nuria Conde*,[1,2] and Ricard Solé†[1,2,6]

[1]*ICREA-Complex Systems Lab, Universitat Pompeu Fabra, 08003 Barcelona, Spain*

[2]*Institut de Biologia Evolutiva (CSIC-UPF), 08003 Barcelona, Spain*

[3]*Departament de Matemàtiques, EPSEB,*
Universitat Politècnica de Catalunya,
Av. Dr. Marañón 44-50, 08028 Barcelona, Spain

[4]*Institut de Matemàtiques de la UPC-BarcelonaTech (IMTech),*
Universitat Politècnica de Catalunya,
Pau Gargallo 14, 08028 Barcelona, Spain

[5]*Centre de Recerca Matemàtica Edifici C, Campus de Bellaterra,*
08193 Cerdanyola del Vallès, Barcelona, Spain

[6]*Santa Fe Institute, 1399 Hyde Park Road, Santa Fe NM 87501, USA*

*Corresponding author: N. Conde (nuria.conde@upf.edu)
†Corresponding author: R. Solé (ricard.sole@upf.edu)

Contents

I. Non-critical gene circuit with congestion 2
A. Deterministic model 2
B. Stochastic dynamics 4

II. Two-gene SOC motif 5
A. Deterministic modelling 7
1. Single fixed point 9
2. Eigenvalues, eigenvectors, and topological information 13
3. Deterministic grounds of SOC 14
B. Stochastic dynamics model 17

III. Experimental Information 21
A. Primers, sequences and plasmids 21
B. Plasmid Map 24
C. FACS analysis 25

IV. References 34

I. NON-CRITICAL GENE CIRCUIT WITH CONGESTION

For the sake of completeness, we provide in this section information on the dynamics, both deterministic and stochastic, of the non-regulated system displayed within the grey frame in Fig. 1, which does not present self-organized criticality (SOC). This non-regulated circuit has been used as a basis to build the SOC motif by coupling another gene inhibiting the synthesis of the GFP (see Fig. 3). In Section II we will describe the SOC motif, focusing on the dynamics allowing for the emergence of sharp stochastic fluctuations providing the SOC behaviour.

A. Deterministic model

The non-regulated system (displayed framed in grey in Fig. 1) involves the constitutive expression of a gene coding for a protein, labeled σ. The degradation of σ takes place both spontaneously and as a result of the cellular proteolytic machinery, driven by means of

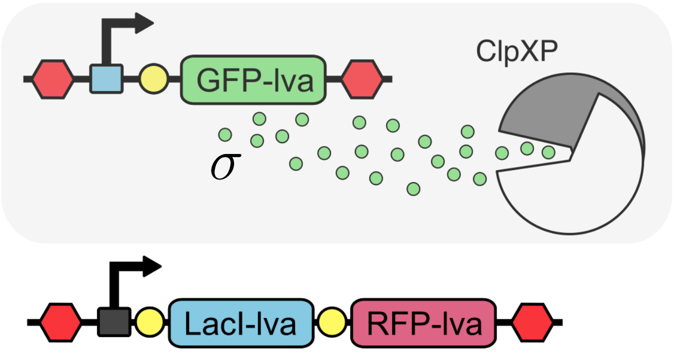

FIG. 1: Schematic representation of the non-regulated system (framed in grey) displaying free and congested phases as a result of a queueing-like process. The GFP protein (labelled σ) is degraded by the ClpXP complex using the recognition tag lva. The complete circuit also includes the degradation of RFP and LacI proteins, that compete with GFP for the degradation complex. When the both modules are coupled (see Fig. 3), SOC dynamics can be achieved.

ClpXP. A simple model describing the dynamics of protein σ follows a deterministic (mean-field) system, given by the differential equation (see also Ref. [1] for further details):

$$\frac{d\sigma}{dt} = \eta - \delta\sigma - \frac{\sigma\rho}{K+\sigma} =: g(\sigma). \tag{1}$$

Here $\eta > 0$ and $\delta > 0$ are the rates of constitutive expression and of spontaneous degradation of protein σ, respectively. Constant $K > 0$ denotes a saturation term of the degradation of σ by ClpXP. Here, following Ref. [1] we define $\rho := \delta_c C$, $C =$ [ClpXP] indicating the (constant) concentration of ClpXP. The equilibrium points of Eq. (1) are obtained from $g(\sigma) = 0$. This system has only a non-negative equilibrium point, given by:

$$\sigma_+ = \frac{1}{2\delta}\left(\eta - \rho - K\delta + \sqrt{(\eta-\rho)^2 + K\delta(2\eta + 2\rho + K\delta)}\right). \tag{2}$$

The stability of this equilibrium can be obtained by linearising Eq. (1), computing

$$\lambda(\sigma_+) = \frac{d}{d\sigma} g(\sigma_+),$$

which gives

$$\lambda(\sigma_+) = -2\,\frac{\delta\left((K\delta + \eta - \rho)\sqrt{K^2\delta^2 + \psi} + K^2\delta^2 + \psi\right)}{\left(K\delta - \rho + \eta + \sqrt{K^2\delta^2 + \psi}\right)^2},$$

where $\psi = 2\,K\,(\rho+\eta)\,\delta + (-\eta+\rho)^2$. Observe that

$$\begin{aligned}
\lim_{K\to+\infty} \sigma_+ &= \lim_{K\to+\infty} \frac{1}{2\delta}\left(\eta-\rho-K\delta+\sqrt{(\eta-\rho)^2+K\delta(2\eta+2\rho+K\delta)}\right)\\
&= \lim_{K\to+\infty} \frac{1}{2\delta}\frac{(\eta-\rho-K\delta)^2-((\eta-\rho)^2+K\delta(2\eta+2\rho+K\delta))}{\eta-\rho-K\delta-\sqrt{(\eta-\rho)^2+K\delta(2\eta+2\rho+K\delta)}}\\
&= \lim_{K\to+\infty} \frac{1}{2\delta}\frac{(K\delta)^2-2K\delta(\eta-\rho)-(K\delta)^2-K\delta(2\eta+2\rho)}{-K\delta-\sqrt{(K\delta)^2+K\delta(2\eta+2\rho)+(\eta-\rho)^2}+\eta-\rho}\\
&= \lim_{K\to+\infty} \frac{1}{2\delta}\frac{2K\delta\,(2\eta)}{K\delta+\sqrt{(K\delta)^2+K\delta(2\eta+2\rho)+(\eta-\rho)^2}+\eta-\rho}.
\end{aligned}$$

Taking the leading terms (in K) in the numerator and denominator, we can conclude that:

$$\sigma_+ \approx \frac{\eta}{\delta}, \tag{3}$$

when K is large enough. On the other hand, observe that similar computations reveal that

$$\lim_{K\to 0} \sigma_+ = \frac{\eta-\rho}{\delta},$$

which leads to a two-phase system defined by a threshold condition $\sigma(\eta) = 0$ for $\eta < \rho$ and $\sigma(\eta) = \sigma_+$ for $\eta > \rho$.

These limit cases are discussed in Ref. [1], where the experimental implementation of this model and some generalisations using several proteins competing for the ClpXP machinery were performed.

The shape of the transitions is summarised in Fig. 2a. In particular, the presence of steep changes in the expression levels as both η and δ_c parameters are varied. Across all this parameter surface, two well-defined domains are observed: the free and congested states.

B. Stochastic dynamics

The stochastic dynamics of this system has been extensively studied [1] and we only summarise some aspects of the kinetics for the sake of comparison with the full SOC model. A stochastic model can be easily build using a set of reactions defined as follows:

$$\varnothing \xrightarrow{W_1} G, \qquad r_1 = +1, \tag{4}$$

$$G \xrightarrow{W_2} \varnothing, \qquad r_2 = -1, \tag{5}$$

$$G + C \xrightarrow{W_3} C, \qquad r_3 = -1, \tag{6}$$

where the reaction rates are defined as follows:

$$W_1 = \eta_1 / \theta, \tag{7}$$

$$W_2 = \delta_1 n_1, \tag{8}$$

$$W_3 = \delta_c n_1 n_3 / (K + n_1). \tag{9}$$

Here n_1 corresponds to the concentrations G/V (σ), where V is the volume at which the reactions occur, and n_3 is the concentration of proteases (C in the mean-field model). By using these stochastic simulation rules, the corresponding distributions of σ levels can be obtained using parameter values before ($\eta < \eta_c$) and after ($\eta > \eta_c$) the free-congested shift. Several of them are displayed in Fig. 2b-g using both linear-log plots (main figures) and log-log scales (insets). Instead of power-law distributions, the distributions close to the boundary are exponential (panels b-d) while they become Gaussian afterwards (panels f-g). The starting point of this transition is visible in the changes of the shape noticeable in Fig.2e, with $\eta \sim \eta_c$. The straight lines of the first region show a single-scale dynamics with an exponential decay distribution of the form $P(s) \sim \exp(-\sigma/\sigma_c)$, and with σ_c rapidly growing as η increases.

II. TWO-GENE SOC MOTIF

In this section we provide a detailed description of the mathematical model describing the dynamics of the two-gene motif. Several analytic and numerical results are detailed, along with a summary of the stochastic implementation. Two components need to be considered here. On the one hand, the basic architecture where both proteins are being degraded by the proteolytic machinery with a negative regulatory feedback. This is the requirement for a SOC system, where the order and the control parameter (σ_1 and σ_2 respectively) interact (see Fig. 1 in the main text). On the other hand, tuning is also used in order to ensure a rapid effect of σ_2 and a proper level of this protein in order to guarantee proper congestion detection. These two tuneable features are introduced in the experimental setup by means of Isopropyl-β-D-thiogalactopyranoside (IPTG) and Arabinose, respectively. Below we comment on how these processes have been implemented in the mathematical model and in the stochastic simulations.

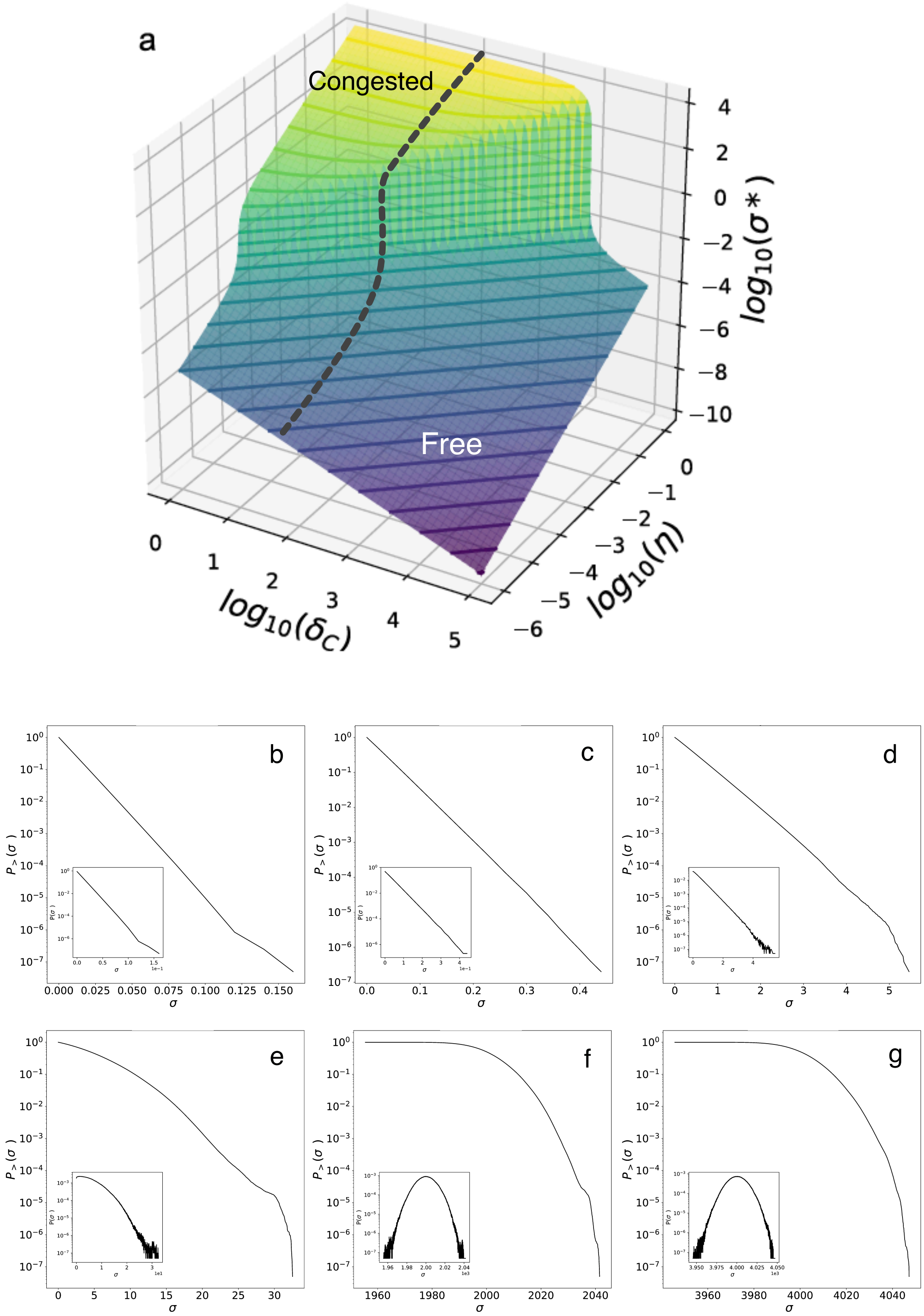

FIG. 2: (a) Equilibrium concentrations of protein σ, defined as σ^*, and displayed against η and δ_C i. e. production and degradation rates, respectively (see main text). One section through the equilibrium surface is displayed on top of this surface, where the transition between free (violet-blue region) and congested (green-yellow region) behaviour occurs as η is changed, when $\eta \approx 0,01$ (this curve is shown in Fig. 1b in the main text). The parameters used are: $\delta = K = 10^{-3}$ and $C = 0.1$ (notice that axes are in logarithmic scale). (b-g) Representative examples of the corresponding distributions of activity are displayed along this section, with $\delta_C = 10^2$ and: $\eta = 0.01$ (b); $\eta = 0.05$ (c); $\eta = 0.095$ (d); $\eta = 0.1$ (e); $\eta = 0.2$ (f); and $\eta = 0.3$ (g).

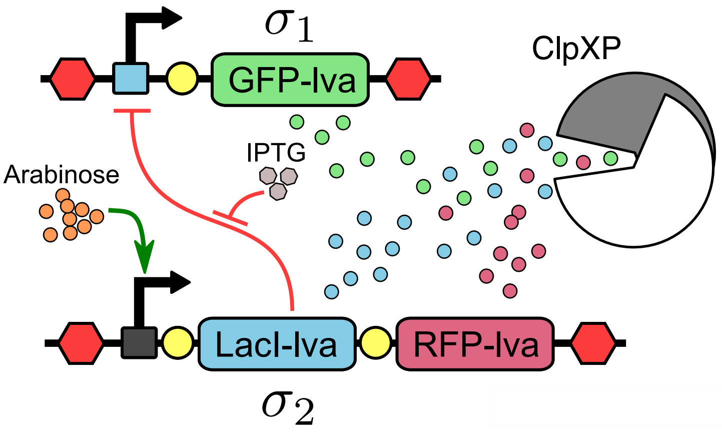

FIG. 3: Schematic representation of the two-gene SOC motif. The expression of GFP (labelled σ_1) is repressed by LacI (σ_2) while both are competing for the degradation complex ClpXP. In the designed genetic circuit, RFP is under the same regulation than LacI in order to monitor the amount of repressor in the system. The production of LacI and RFP is induced by Arabinose, whereas the inhibition strength of LacI can be reduced by the addition of IPTG. In our model, both regulations are represented by $f(\sigma_2)$ and μ, respectively.

A. Deterministic modelling

The model describing the feedback loop that provides the SOC architecture is shown in Fig. 3, and its dynamics can be studied with the next set of equations:

$$\begin{cases} \dfrac{d\sigma_1}{dt} = f(\sigma_2) - \delta_1\,\sigma_1 - \sigma_1\Gamma(\sigma_1,\sigma_2), \\ \\ \dfrac{d\sigma_2}{dt} = \eta_2 - \delta_2\,\sigma_2 - \sigma_2\Gamma(\sigma_1,\sigma_2). \end{cases} \tag{1}$$

The first differential equation has a nonlinear response function given by:

$$f(\sigma_2) = \frac{\eta_1}{\theta + \mu^2\,\sigma_2^2}.$$

The function $\Gamma(\sigma_1,\sigma_2)$ is defined as:

$$\Gamma(\sigma_1,\sigma_2) = \frac{\delta_c C}{K + \sigma_1 + \sigma_2}. \tag{2}$$

We assume all the parameters to be positive and arrange them in the parameter vector

$$\mathbf{\Lambda} := (\eta_1,\eta_2,\theta,\delta_1,\delta_2,\delta_c,C,K,\mu^2).$$

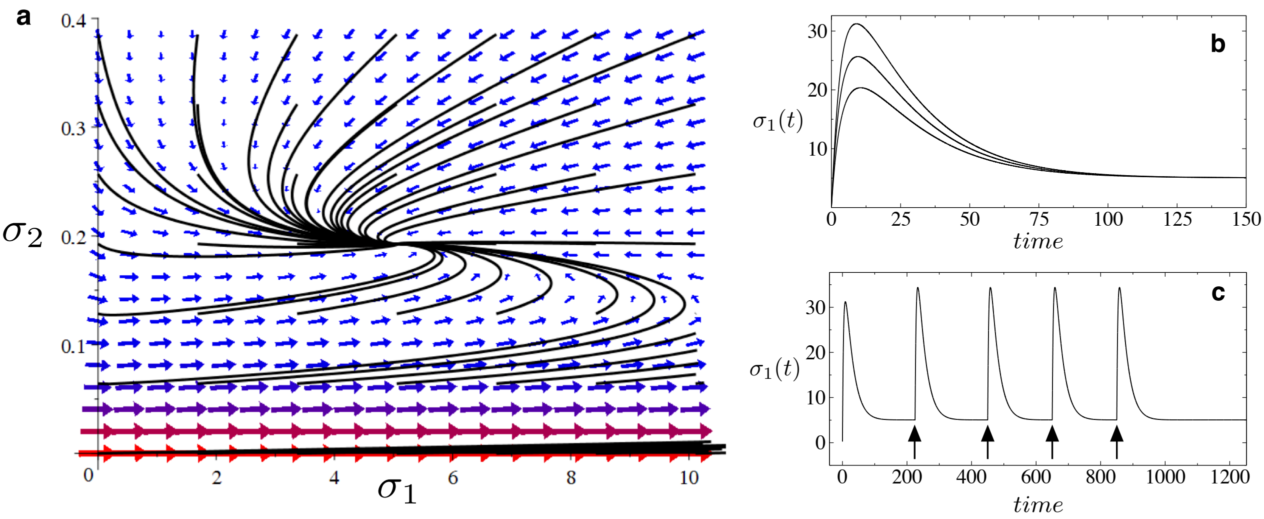

FIG. 4: (a) Phase portrait with several orbits (black curves) obtained with $\eta_2 = 10^{-2}$ (near the peak in the coefficient of variation CV for σ_1, see Fig. 4 in the main manuscript). The colour and the size of the arrows denote the magnitude of the vector field: red, long, thick arrows indicate larger magnitudes and thus faster dynamics. (b) Time series also using $\eta_2 = 10^{-2}$ obtained from three different initial conditions setting $\sigma_1(0) = 0.25$ and (from top to down): $\sigma_2(0) = 10^{-3}$, $\sigma_2(0) = 10^{-2}$, $\sigma_2(0) = 0.02$. (c) Time dynamics using the initial condition $\sigma_1(0) = 0.25$, $\sigma_2(0) = 10^{-3}$, and introducing several perturbations (indicated with the arrows) once the orbit has achieved the attractor. The perturbations, applied four times in the dynamics computed numerically, have been introduced diminishing the value of σ_2 from its equilibrium value to $\sigma_2 = 10^{-4}$.

In our analyses, we will use (unless otherwise stated) the default values

$$\mathbf{\Lambda}_0 := (10^{-2}, \eta_2, 10^{-3}, 5 \cdot 10^{-2}, 5 \cdot 10^{-2}, 10^{-1}, 10^{-1}, 10^{-3}, 1).$$

Parameter η_2 will be generally set as a free parameter. The solutions of Eqs. (1) have been computed with the fourth-order Runge-Kutta method with step size $\delta t = 10^{-2}$. Below, several analytic and numerical results are provided to characterize the qualitative and quantitative properties of the SOC motif dynamics. An illustration of the complex behaviour of the model close to the SOC states (as discussed in the main document) is shown in Fig. 4, where the phase portrait and some time series associated to it are displayed. A specially interesting behavioural pattern is shown in Fig. 4c, where the dynamics of σ_1 is displayed under small perturbations of σ_2 that trigger fast spikes followed by relaxation. This phenomenon, as we discuss in the main text, is a key feature of the inherent stochastic dynamics tied to the critical state of SOC.

1. Single fixed point

Here we show that the SOC motif model reveals a single attractor within the meaningful domain defined by the non-negative concentration space, namely

$$\Omega_1 := \{(\sigma_1, \sigma_2) : \sigma_1 \geq 0, \sigma_2 \geq 0\}.$$

Theorem 1. *System (1) has a unique equilibrium in Ω_1 which is globally asymptotically stable.*

We first study the conditions that the the equilibrium points of the system must satisfy. From $\dfrac{d\sigma_2}{dt} = 0$ in (1), we obtain:

$$\sigma_1 = \sigma_2 \frac{\delta_c C}{\eta_2 - \delta_2 \sigma_2} - K - \sigma_2 =: \Sigma(\sigma_2). \tag{3}$$

Note that $\Sigma(\sigma_2)$ is defined whenever $\sigma_2 \neq \eta_2/\delta_2$. When $\sigma_2 = \eta_2/\delta_2$,

$$\frac{d\sigma_2}{dt} = -\frac{\eta_2 \delta_c C}{\delta_2(K + \sigma_1) + \eta_2},$$

which never vanishes. Therefore, it is not a constraint since no equilibrium points will satisfy $\sigma_{2,eq} = \eta_2/\delta_2$. We extend this comment below in the example devoted to the parameter set $\mathbf{\Lambda}_0$, see also Fig.5.

Substituting $\sigma_1 = \Sigma(\sigma_2)$ into the first equation of (1), we get:

$$\left\{ \begin{aligned} \frac{d\sigma}{dt} &= \eta_2 + \eta_1/(\theta + \mu^2 \sigma_2^2) - \delta_1 \Sigma(\sigma_2) - (\delta_2 - \delta_1)\sigma_2 - \Sigma(\sigma_2)(\delta_c C)/(K + \Sigma(\sigma_2)) \\ &= \eta_2 + \eta_1/(\theta + \mu^2 \sigma_2^2) - \sigma_2 \frac{\delta_1 \delta_c C}{\eta_2 - \delta_2 \sigma_2} + \delta_1 K - (\delta_2 - \delta_1)\sigma_2 - \delta_c C + K \frac{\eta_2 - \delta_2 \sigma_2}{\sigma_2} \\ &= \frac{P_5(\sigma_2)}{Q_4(\sigma_2)}, \end{aligned} \right. \tag{4}$$

where $P_5(\sigma_2) = \sum_{j=0}^{5} \alpha_j \, \sigma_2^j$ with

$$
\begin{aligned}
\alpha_5 &= (\delta_2 - \delta_1)\, \delta_2\, \mu^2, \\
\alpha_4 &= -\left(-C\delta_c + \delta_1 K + \eta_2\right)\, \mu^2 \delta_2 - (\delta_2 - \delta_1)\, \mu^2 \eta_2 - \delta_1 \delta_c C\, \mu^2 + K {\delta_2}^2\, \mu^2, \\
\alpha_3 &= \left(-C\delta_c + \delta_1 K + \eta_2\right)\, \mu^2 \eta_2 + (\delta_2 - \delta_1)\, \theta\, \delta_2 - 2\, K \eta_2 \delta_2\, \mu^2, \\
\alpha_2 &= -\left(-C\delta_c + \delta_1 K + \eta_2\right) \theta\, \delta_2 - (\delta_2 - \delta_1)\, \theta\, \eta_2 - \eta_1 \delta_2 - \delta_1 \delta_c C \theta + K {\eta_2}^2\, \mu^2 + K {\delta_2}^2 \theta, \\
\alpha_1 &= \left(-C\delta_c + \delta_1 K + \eta_2\right) \theta\, \eta_2 + \eta_1 \eta_2 - 2\, K \eta_2 \delta_2 \theta, \\
\alpha_0 &= K\, \theta\, {\eta_2}^2;
\end{aligned}
$$

and

$$Q_4(\sigma_2) = \left(\theta + \mu^2\, {\sigma_2}^2\right) (\eta_2 - \delta_2 \sigma_2)\, \sigma_2.$$

Therefore, to find the equilibria of the system we must solve the degree five polynomial equation $P_5(\sigma_2) = 0$. Observe also that for $\delta_1 = \delta_2$ (satisfied in our distinguished parameter set $\mathbf{\Lambda}_0$), P_5 becomes a degree four polynomial since $\alpha_5 = 0$. Without making any computation, then, it seems very likely that it will have more than one zero. However, next results ensure that we have only one positive root.

Lemma 1. *For any set of positive parameters $\mathbf{\Lambda}$, there exists a constant $M_0 = M_0(\mathbf{\Lambda})$ such that any region $\Omega_{1,M}$ bounded by the two positive semiaxes and the segment of the line defined by $\sigma_1 + \sigma_2 = M$ belonging to Ω_1 is positively invariant by the flow of (1).*

Proof. We prove Theorem 1 by observing that the divergence of system (1) is

$$
\begin{aligned}
\mathrm{div} X &= -\delta_1 - \delta_2 - 2\, \frac{\delta_c C}{K + \sigma_1 + \sigma_2} + (\sigma_1 + \sigma_2) \frac{\delta_c C}{(K + \sigma_1 + \sigma_2)^2} \\
&= -(\delta_1 + \delta_2) - \frac{\delta_c C}{(K + \sigma_1 + \sigma_2)^2} (2K + \sigma_1 + \sigma_2),
\end{aligned}
\tag{5}
$$

which is negative provided that both the variables and the parameters are positive. This property, together with the existence of the invariant region provided by Lemma 1, implies that the equilibrium is unique and globally asymptotically stable for (σ_1, σ_2) in Ω_1. □

Equilibrium points and dynamics for the parameter set $\mathbf{\Lambda}_0$.
In Fig. 5 we show the position of equilibria for the parameter set $\mathbf{\Lambda}_0$ letting η_2 as a free parameter. Going back to the domain of definition of $\Sigma(\sigma_2)$ discussed above (see equation (3) and Fig.5), we can appreciate that $\sigma_{2,eq}$ tends to zero as η_2 tends to zero and, moreover,

it is asymptotically tangent to a straight line, say $\sigma_{2,eq} \sim \kappa\,\eta_2$. Imposing this condition on $P_5(\sigma_2)$, we get

$$\begin{aligned} P_5(\kappa\,\eta_2) \; &= \eta_2^2\,(-0.01\kappa^3\eta_2^2 - 5\times 0^{-4}\,\kappa^2 + 0.99\times 10^{-2}\,\kappa - 5\times 10^{-2}\,\kappa^4\eta_2^3 + \kappa^3\eta_2^3 - 5\times 10^{-5}\,\kappa^2\eta_2 \\ &\quad +10^{-3}\,\kappa\,\eta_2 + 10^{-3}\,\kappa^2\eta_2^2 + 10^{-6})\,. \end{aligned}$$

The expression inside the parenthesis only vanishes if $\kappa \approx -0.1\times 10^{-3}$, which does not make sense biologically speaking, and $\kappa \approx 19.9$. Thus, we get that $\sigma_{2,eq} \sim \kappa^*\,\eta_2$ when $\eta_2 \to 0^+$, with $\kappa^* := 19.9$. Consequently,

$$\lim_{\eta_2\to 0^+,\,\sigma_{2,eq}=\kappa^*\eta_2} \sigma_{1,eq} = \lim_{\eta_2\to 0^+} \Sigma(\kappa^*\eta_2) = \lim_{\eta_2\to 0^+} \kappa^*\,\frac{\delta_c\,C}{1-\delta_2\,\kappa^*} - K = 199.8,$$

as we can appreciate in Fig.5, using the parameter values from the set $\mathbf{\Lambda}_0$.

Another way to analyze the effect of introducing the LacI repressor protein (σ_2) is visualizing the changes in the separation between free and congested phases as we increase the amount of production, leveraged by η_2, of this inhibitor. In Fig. 6, we extend the curve of equilibrium points of the 1D system (1) given in Fig. 2b, which shows the dependence of the equilibrium point of the green fluorescent protein (σ_1) on its production rate (η_1). The curves in Fig. 6 show four instances of the log-log $\sigma_{1,eq}$ versus η_1 plot for four different values of $\sigma_{2,eq}$ (0, 0.02, 0.2 and 2), which correspond approximately to $\eta_2 = 0,\ 10^{-3},\ 10^{-2}$ and 0.1, respectively. That is, the $\sigma_2 = \sigma_{2,eq}$ value assigned to each curve is the second component of the equilibrium of system (1) for the respective η_2 value; this σ_2 constant value is then plugged into the first equation of (1) and the curve is obtained by isolating σ_1 in terms of η_1. All other parameters are those of the parameter set $\mathbf{\Lambda}_0$, except for $\delta_C = 50$ (to agree with the 1D case). Observe that the curve for $\eta_2 = \sigma_{2,eq} = 0$ (red) corresponds to that of Fig. 2b.

We observe that the levels of the σ_1 equilibria of the free and congested phases approach each other as η_2 increases, thus eliminating the all-or-none paradigm of the 1D model and favoring the possibility of the dynamics to develop close to the phase boundary. The std/mean peak described in Fig. 2d (main paper) would correspond to a situation close to the blue curve ($\sigma_{2,eq} = 0.2$) of Fig. 6.

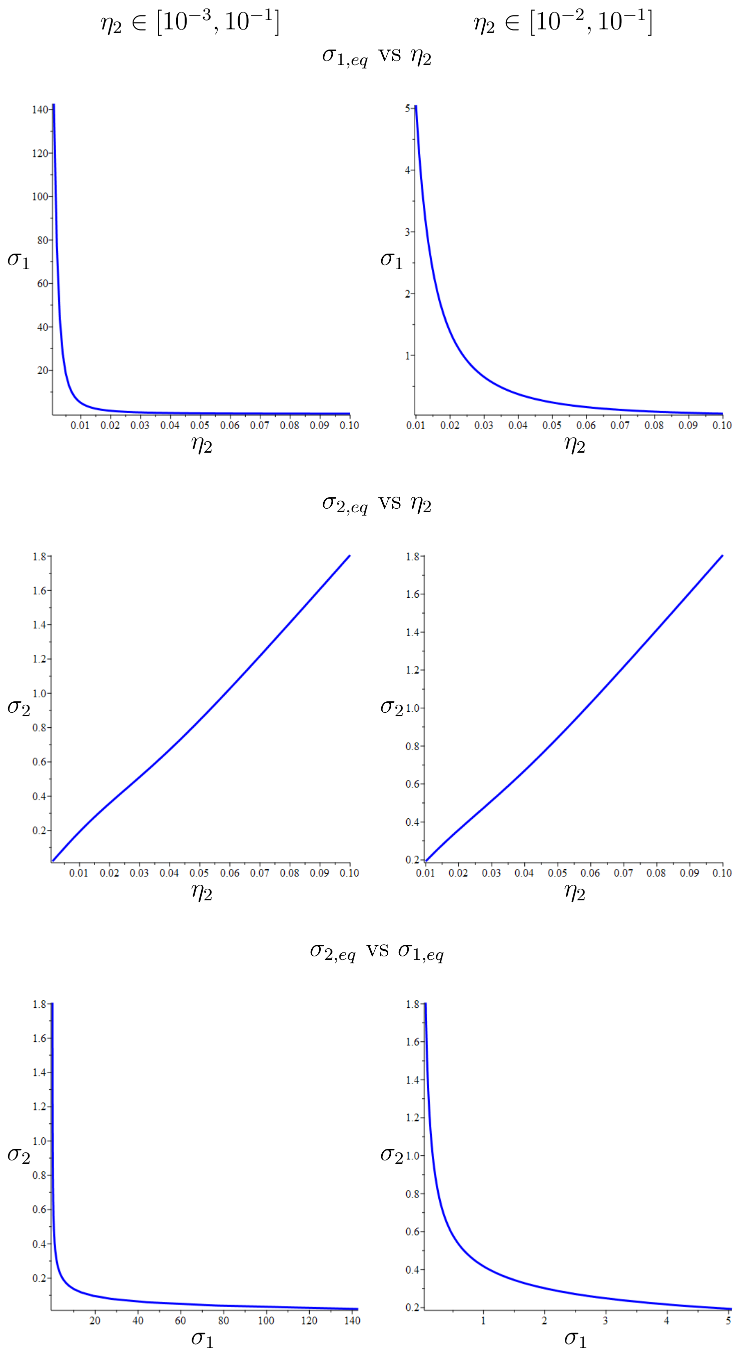

FIG. 5: Equilibrium points in terms of η_2. The upper (respectively, middle) panels show ($\sigma_{1,eq}$ (respectively, $\sigma_{2,eq}$)) versus η_2. Bottom panels show $(\sigma_{1,eq}, \sigma_{2,eq})$; the curve is parameterized by η_2, which increases from the lowest right point to the highest left point. For the left panels, $\eta_2 \in [10^{-3}, 10^{-1}]$; in the right panels we show a zoom for $\eta_2 \in [10^{-2}, 10^{-1}]$.

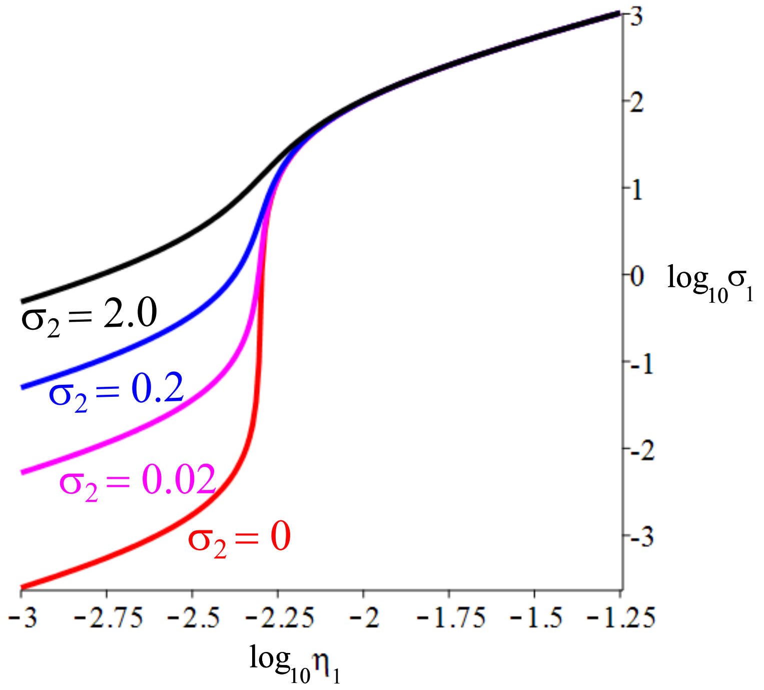

FIG. 6: Log-log plot of the equilibrium first coordinates in terms of η_1 for system (1). As described in the text, the four curves correspond to different values of $\sigma_2 = \sigma_{2,eq}$, which vanish the second equation of (1) for four corresponding values of η_2, which are respectively close to $\eta_2 = 0$, 10^{-3}, 10^{-2} and 0.1.

2. Eigenvalues, eigenvectors, and topological information

The Jacobian of system (1) is

$$DX = \begin{pmatrix} -\delta_1 - \dfrac{\delta_c C}{K + \sigma_1 + \sigma_2} + \sigma_1 \Gamma(\sigma_1, \sigma_2) & \dfrac{-2\,\eta_1\,\mu^2\,\sigma_2}{(\theta + \mu^2\,\sigma_2^2)^2} + \sigma_1 \Gamma(\sigma_1, \sigma_2) \\ \\ \sigma_2\,\Gamma(\sigma_1, \sigma_2) & -\delta_2 - \dfrac{\delta_c C}{K + \sigma_1 + \sigma_2} + \sigma_2 \Gamma(\sigma_1, \sigma_2) \end{pmatrix}. \quad (6)$$

From now on, we will fix the set of values except for η_2, and we are going to explore the behaviour of the system in terms of this parameter. For the set of parameters $\mathbf{\Lambda}_0$ (we recall that η_2 is free), we have computed the eigenvalues using a computer algebra system from the expression of the Jacobian (6) and then approximated them numerically.

We first explored the range $\eta_2 \in [0, 1]$, in which (as shown above) the equilibrium is always an attractor. We observe an interesting feature around $\eta_2 \approx 0.15$: the switch from an attracting focus to an attracting node, see Fig. 7(a) (a less noticeable switch from an attracting node to an attracting focus occurs at $\eta_2 \approx 0.17 \times 10^{-4}$). If the reported SOC phenomenon was local, this could be a landmark for the appearance of stochastic phenomena

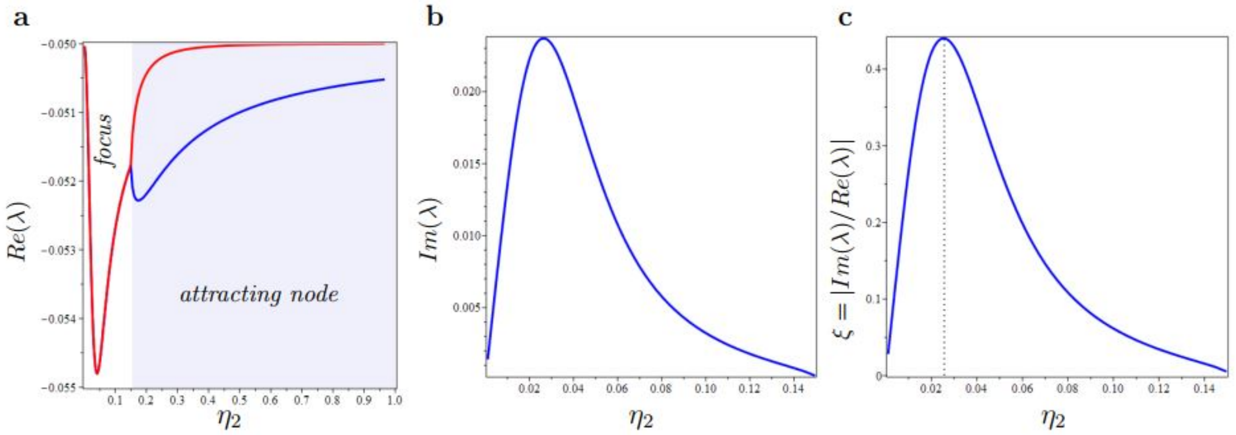

FIG. 7: (a) Real part α of the eigenvalues ($\lambda = \alpha \pm \beta i$) against $\eta_2 \in [0, 1]$. When $\eta_2 = \eta_{2,sw} \approx 0.1497$, the fixed point changes from an attracting focus to an attracting node (grey area). (b) Imaginary part α of the eigenvalues in the focus region, that is, for $\eta_2 < \eta_{2,sw}$. Interestingly, a maximum is observed (c, dashed line) near $\eta_2 \approx 0.0254$ in the quotient ξ between the imaginary and the real part of the eigenvalues (in absolute value), revealing a possible landmark of the (optimal) SOC state found in the stochastic model.

because perturbation around a focus are phase dependent in contrast with nodes. However, despite of the possible relevance of the focus-to-node (or vice versa) switch, there is little difference between the focuses and the nodes close to the switching point because the rotation of the focus is very weak. Indeed, a rotation index for a focus with eigenvalues $\alpha \pm \beta i$ can be captured by the quotient $\xi := |\beta/\alpha|$ (for a pure rotation, the eigenvalue is imaginary and so $\xi \to \infty$ whereas, near to the focus-to-node β, approaches zero and so $\xi \to 0$). We represent this quotient in the η_2-range in Fig. 7(c)). This figure indicates that close to $\eta_2 \sim O(10^{-2})$, the imaginary-to-real ratio ξ is maximal, so in this spot we have maximal rotation near the equilibrium.

3. Deterministic grounds of SOC

The peak in the coefficient of variation (CV) of σ_1 underlying the SOC scenario in the stochastic system (see Fig.2d, main paper) has its grounds on the deterministic dynamics given by Eqs. (1), which captures the sensitivity of σ_1 in different regions of the parameter space. We explore the combination of two factors: the slope of the nullclines and the magnitude of the derivative $d\sigma_1/d\sigma_2$ in the phase space. A transformation of this derivative, intended to enhance the differences in magnitude, is shown in Fig. 8. More precisely, we represent

$$\Delta\sigma_{12} := \frac{1}{2}\text{sgn}(w)\ln(1 + w^2), \tag{7}$$

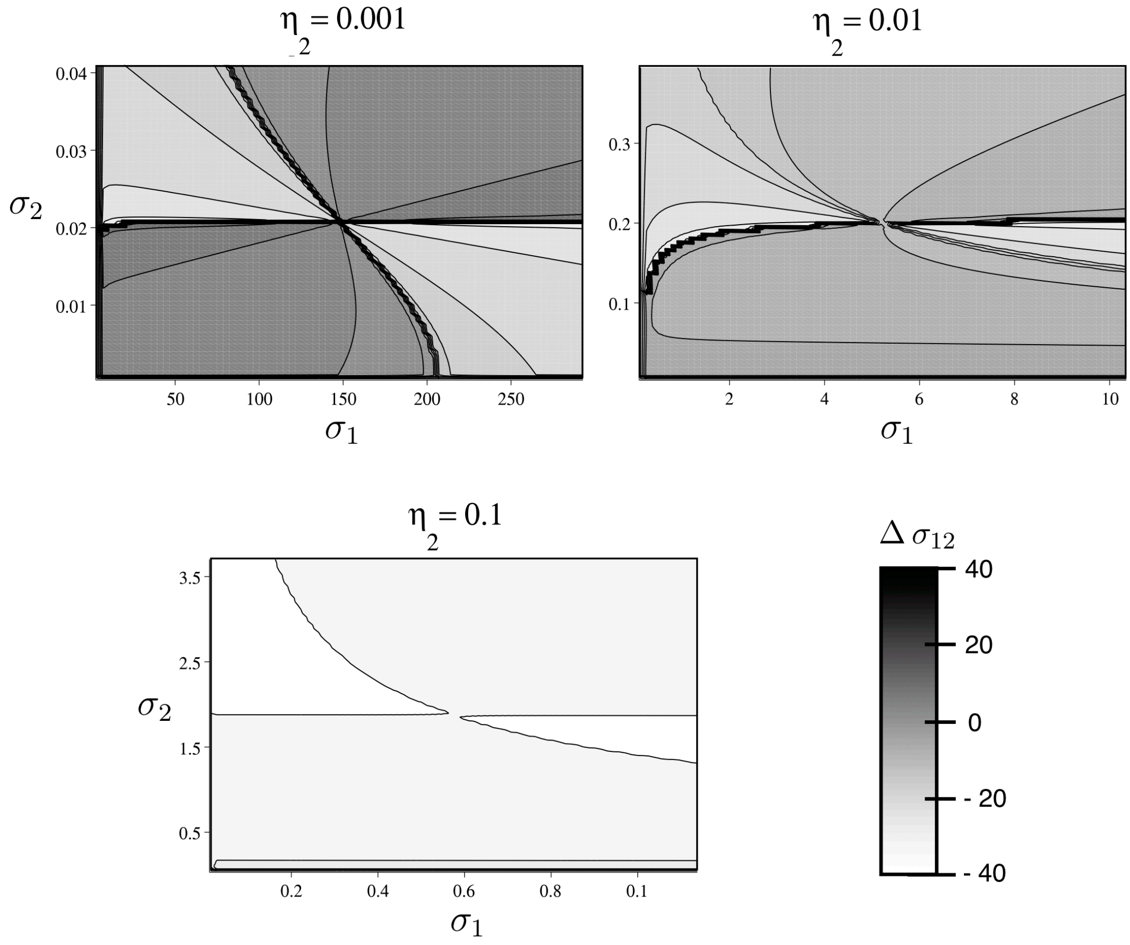

FIG. 8: Graphical representation of $\Delta\,\sigma_{12}$, see (7), according to system (1) near the CV peak observed in the stochastic simulations for σ_1. Every panel is centered at the equilibrium point $(\sigma_1(\eta_2),\, \sigma_2(\eta_2))$, that is, it displays the phase portrait on $[0\,,\,2\,\sigma_1(\eta_2)] \;\times\; [0\,,\,2\sigma_2\,(\eta_2)]$. White and black gradient indicates the values of $\Delta\,\sigma_{12}$ from the most negative to the most positive values in all panels, respectively.

where $w := \frac{d\sigma_1}{d\sigma_2}$, for $\eta_2 \in [10^{-3}, 10^{-1}]$.

For values of η_2 under the CV peak (left), a relatively high (darker color) $\Delta\sigma_{12}$ values are observed but also a high-slope vertical nullcline. For values of η_2 above the CV peak (right), a low-slope vertical nullcline is observed but a relatively low (whiter) $\Delta\sigma_{12}$ values. Values of η_2 near to the CV peak (center) present an optimal balance between the magnitude of $\Delta\sigma_{12}$ (dark enough) and a low-slope vertical nullcline. This balance allows that stochastic perturbation cause a strong effect without reaching the vertical nullcline, which turns into large excursions in the σ_1 variable.

Self-organised criticality involves that the system has a critical point as attractor, meaning that the system poises itself close to the transition boundary. The canonical example of this is the sand or rice pile: as the system enters into the dynamical regime where

avalanches are generated, one observation concerning the long-term dynamics is that the control parameter will exhibit fluctuations close to its critical value. In this case, it would be the angle of repose θ_c (as discussed in the main manuscript, see figure 1c). Each time an avalanche occurs, the slope θ can decrease below the critical θ_c control parameter, which will then start to grow back towards θ_c as new sand grains are slowly added to the pile.
To illustrate this phenomenon within the context of the system studied here, we have monitored how the control parameter, as described in terms of an inhibition function, changes in time. Specifically, for the parametric scenario where SOC has been identified (fixing parameters as in Fig. 2e in the main manuscript), this can bee done by re-writing our original set of equations as:

$$\frac{d\sigma_1}{dt} = \hat{\eta}_1 - \delta_1 \sigma_1 - \sigma_1 \frac{\delta C}{K + \sigma_1 + \sigma_2},$$

with

$$\hat{\eta}_1 := f(\sigma_2) = \frac{\eta_1}{\theta + \mu^2 \sigma_2^2}. \tag{8}$$

This is possible provided (as it occurs in our SOC design) that a fast dynamics is involved for σ_2. Using this definition, we can As expected, the order parameter (the activity of our system) shows a flat region at low values of $\hat{\eta}_1$ (i.e., $\hat{\eta}_1 \lesssim 0.02$). At $\hat{\eta}_1 \approx 0.02$ the system enters into the congested phase, where σ_1 rapidly grows (solid green line in Fig. 9). To illustrate the SOC behavior, we have monitored the time evolution of $\hat{\eta}_1(t)$.
In a SOC regime, $\hat{\eta}_1(t)$ should constantly move towards the boundary between the free and congested phases. This is exactly what we obtain. Figure 9 shows how this dynamical parameter is indeed evolving towards this transition value. Specifically, we have computed the effective repression driven by $\hat{\eta}_1(t)$ monitoring the value of this dynamical parameter using the stochastic realisation shown in Fig. 2e in the main manuscript (the one located on the peak in the CV); that is, the case with criticality and power-law distributions obtained for $\eta_2 = 10^{-2}$ and $\mu = 1.5$. As shown in Fig. 9, the dynamical parameter is always close to $\hat{\eta}_1 \approx 0.02$. In other words (as with the angle of the sandpile), due to the fluctuations of the system at the critical point, $\hat{\eta}_1(t)$ is constantly bouncing back and forth around the transition point associated to the original, non-regulated system. Figure 9 displays this behaviour by showing the probability of the dynamical parameter taking the values of $\hat{\eta}_1$ (labeled $P(\hat{\eta}_1)$).
What about other parameter domains? How do they influence the presence and robustness of the resulting activity distributions? Up to now, we have focused on a single parameter set to study SOC. To check how robust is SOC to the production rates of both proteins,

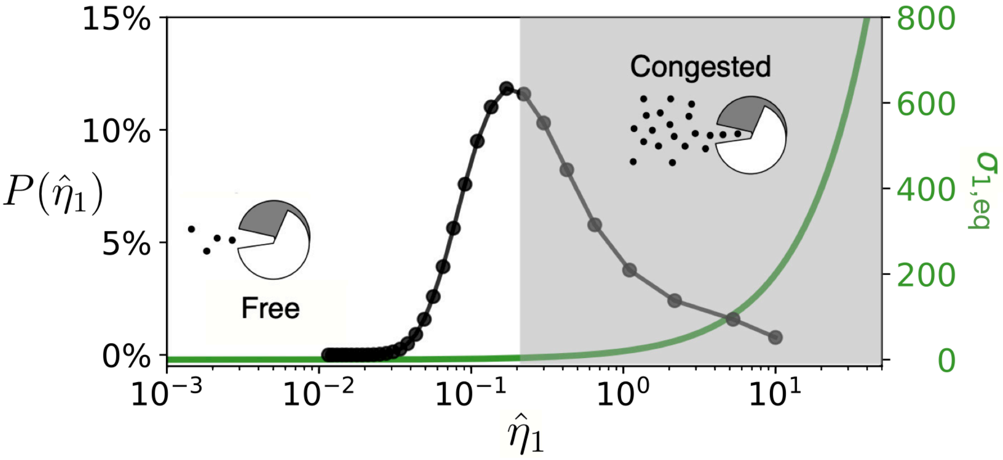

FIG. 9: The spontaneous tendency towards the critical state is summarized here by computing the probability $P(\hat{\eta}_1)$ (the control parameter, giving by the effective inhibition of σ_1) against $\hat{\eta}_1(t)$. As expected from the operation of the SOC motif, this effective control parameter is observed close to the Free (white) - Congested (gray) boundary of the original, non-regulated system, with a maximum located close to $\hat{\eta}_1 \approx 0.02$. We also plot the stationary concentration of σ_1 (order parameter) as a function of $\hat{\eta}_1$ (control parameter) for the parameter values from Fig. 2e in the main manuscript.

we have tuned η_1 and η_2 within the same range, keeping all other parameters fixed as in Fig. 2(e). The results are displayed in Figs. 10-11. The simulations show that the SOC behaviour remains for different values of η_1 within one order of magnitude, and the system is able to poise itself around the transition boundary (Fig. 10(a)). As expected, for all the values of η_1 analysed we found power-law behaviour and the characteristic time series with spikes (Fig. 10(b)-(c)). However, when we changed η_2 (the parameter associated to the tuneable pBAD promoter in our experiments) different behaviours are found, among them some simulations displaying the SOC properties when the value of η_2 is around $\eta_1 = 10^{-2}$ (see Fig. 11).

B. Stochastic dynamics model

We introduce a Markovian stochastic model now consisting of 6 processes involved in the synthetic SOC motif, with A and B denoting the molecules of σ_1 and σ_2, respectively, with

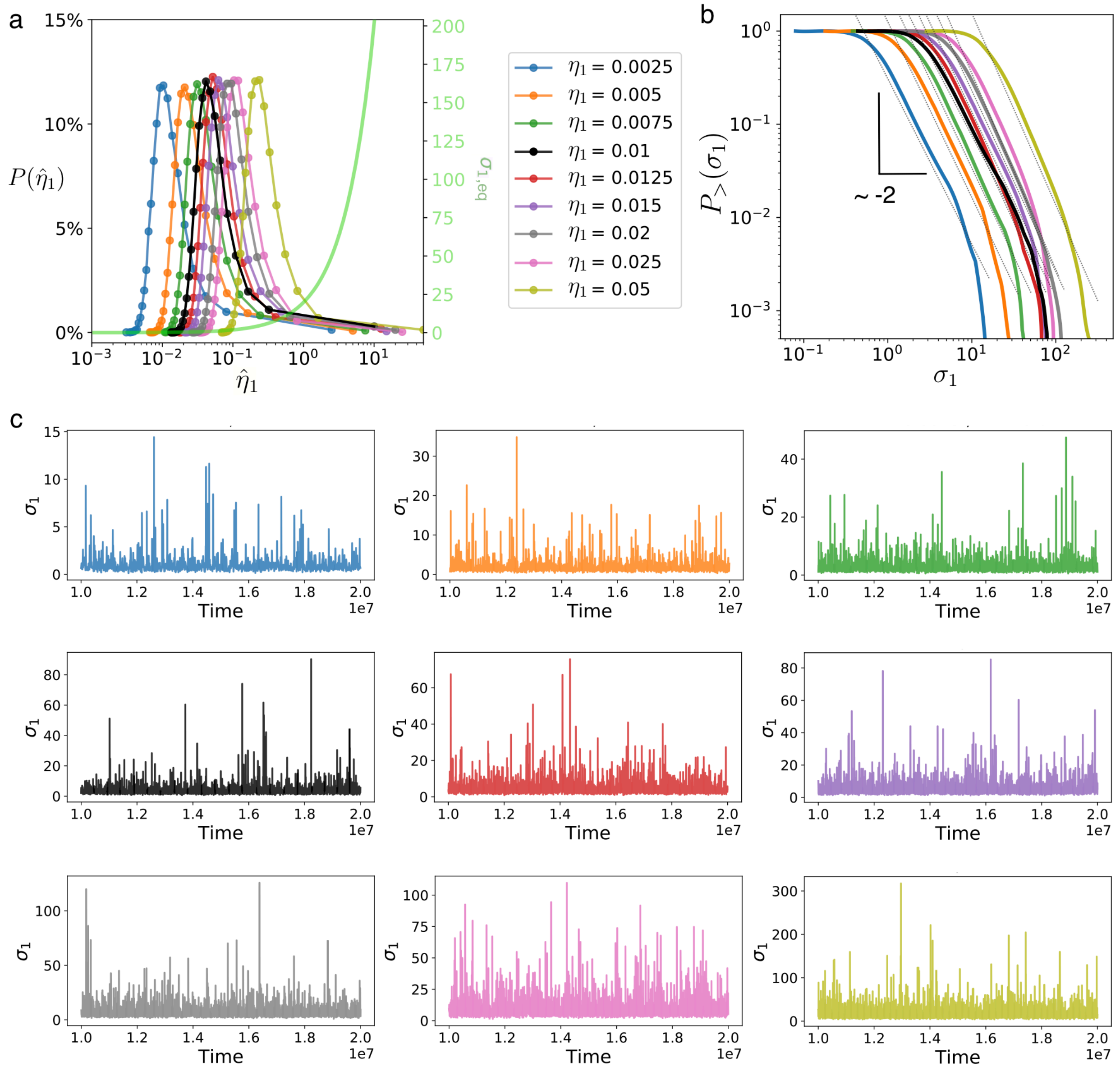

FIG. 10: Robust bursty, scale-free behavior of the SOC motif system under varying η_1 values, keeping all other parameters as in Fig. 2(e), with $\eta_2 = 10^{-2}$. (a) Varying η_1 between 2.5×10^{-3} and 0.05, the dynamical parameter $\hat{\eta}_1(t)$ mainly stays close to the transition boundary. The power-law behaviour expected near the transition boundary is shown for the same values of η_1 (displayed with the same colours) in panel (b). (c) Time series for each η_1 value (also using the same coloring). Notice that all the time series appear qualitatively similar i.e., low values of $\sigma_1(t)$ with spikes.

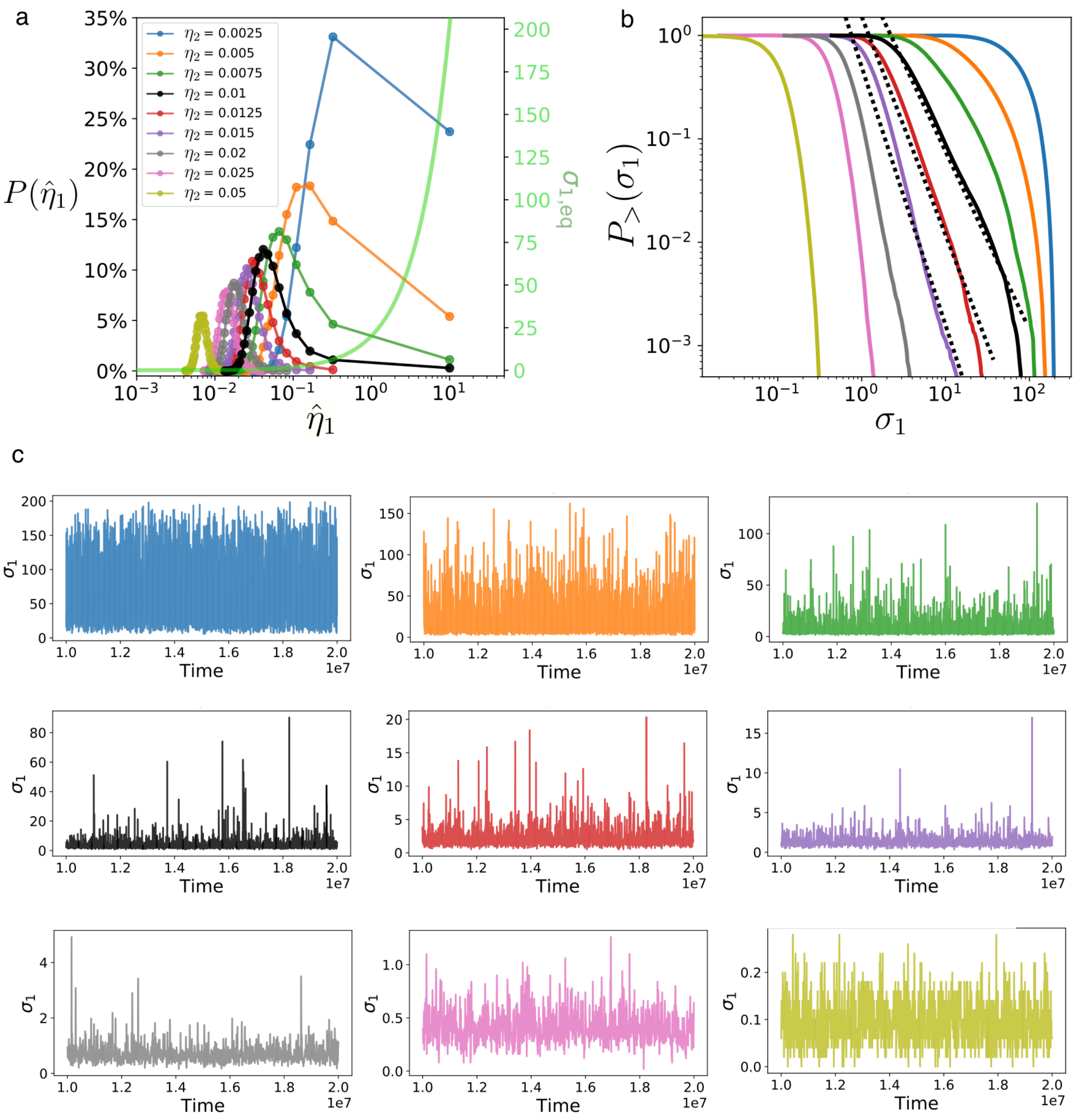

FIG. 11: Same as in Fig. 10 now changing η_2 and fixing $\eta_1 = 10^{-2}$. The values of $\eta_2 = 0.0125$ (red line) and $\eta_2 = 0.015$ (violet line) still retain power-law distributions with exponents close to -2: ~ -2.11 (red line) and ~ -2.3 (violet line) (as panel (b) shows). The other values of η_2 departing from the tuned approach close to the transition boundary do not have this characteristic scaling exponent. The time series for those cases with clear power-law distributions keep showing the spiky dynamics.

C being the concentration of ClpXP and V the volume (system's size):

$$\varnothing \xrightarrow{W_1} A, \qquad r_1 = +1, \tag{9}$$

$$A \xrightarrow{W_2} \varnothing, \qquad r_2 = -1, \tag{10}$$

$$A + C \xrightarrow{W_3} C, \qquad r_3 = -1, \tag{11}$$

$$\varnothing \xrightarrow{W_4} B, \qquad r_4 = +1, \tag{12}$$

$$B \xrightarrow{W_5} \varnothing, \qquad r_5 = -1, \tag{13}$$

$$B + C \xrightarrow{W_6} C, \qquad r_6 = -1, \tag{14}$$

Here r_i corresponds to the stoichiometric value of each reaction. The propensities (transition rates) for this system are modelled as follows (using the Law of Mass Action):

$$\begin{aligned}
W_1 &= \eta_1 / (K + \mu^2 n_2^2), \\
W_2 &= \delta_1 n_1, \\
W_3 &= \delta_c n_1 n_3 / (K + n_1 + n_2) \\
W_4 &= \eta_2, \\
W_5 &= \delta_2 n_2, \\
W_6 &= \delta_c n_2 n_3 / (K + n_1 + n_2),
\end{aligned}$$

where n_1 and n_2 correspond to the concentrations A/V (σ_1) and B/V (σ_2), and V is the volume where the reactions occur. Finally, n_3 is the concentration of ClpXP. The above reactions have been implemented with the Gillespie algorithm [2–4].

III. EXPERIMENTAL INFORMATION

A. Primers, sequences and plasmids

Name	Description	Sequence
Fw-EcoRIpBAD30Lac	*EcoRI* enzyme restriction site, *pBAD* promoter, *RBS*30 and starting *LacI* sequence	catcatgaattcacattgattatttgcacggcgtcacactttgctatgccatagcatttttatccataagattagcggatcctacctgacgctttttatcgcaactctctactgtttctccataccgtttttttgggctagcattaaagaggagaaaatggtgaatgtgaaaccagtaacg
Rv-lva30ppiRFP	Ending *LacI* sequence, *RBS*30 and start RFP sequence (reverse sequence)	gctgcaaacgacgaaaactacgctttagtagcttaatctagtattaaagaggagaaatctagtatggcttcctccgaagacgttatcaaagag
Fw-lva30ppiRFP	Ending *LacI* sequence, *RBS*30 and start RFP sequence	ctctttgataacgtcttcggaggaagccatactagatttctcctctttaatactagattaagctactaaagcgtagttttcgtcgtttgcagc
Rv-lvaterXbal	*lva* degradation tag and *XbaI* restriction enzyme site (reverse sequence)	atgatgtctagatataaacgcagaaaggcccacccgaaggtgagccagtgtgattattaagctactaaagcgtagttttcgtc
Fw-SpeIplacIiQ34GFP	SpeI restriction site, *pLac* promoter, *RBS*34 and starting GFP sequence	catcatactagttcacactggctcaccttcgggtgggagcggcatgcatttacgttgacaccacctttcgcggtatggcatgatagcgcccggtctagtaaagaggagaaatctagtatgcgtaaaggagaagaact
Rv-lvaPstI	*lva* degradation tag and *PstI* restriction enzyme site (reverse sequence)	atgatgctgcagttattaagctactaaagcgtagttttcgtc

TABLE I: List of primers used in the experiments performed in this article.

Name	Description	Sequence
pBlueScript II SK(+)	Basic cloning vector. With Amp	
GFP-lva (BBa_K145015)	Green fluorescence protein with degradation tag	atgcgtaaaggagaagaacttttcactggagttgtcccaatt cttgttgaattagatggtgatgttaatgggcacaaattttct gtcagtggagagggtgaaggtgatgcaacatacggaaaac ttacccttaaatttatttgcactactggaaaactacctgttcc atggccaacacttgtcactactttcggttatggtgttcaatgc tttgcgagatacccagatcatatgaaacagcatgactttttc aagagtgccatgcccgaaggttatgtacaggaaagaactat atttttcaaagatgacgggaactacaagacacgtgctgaag tcaagtttgaaggtgatacccttgttaatagaatcgagttaa aaggtattgattttaaagaagatggaaacattcttggacac aaattggaatacaactataactcacacaatgtatacatcatg gcagacaaacaaaagaatggaatcaaagttaacttcaaaa ttagacacaacattgaagatggaagcgttcaactagcagac cattatcaacaaaatactccaattggcgatggccctgtcctt ttaccagacaaccattacctgtccacacaatctgccctttcg aaagatcccaacgaaaagagagaccacatggtccttcttga gtttgtaacagctgctgggattacacatggcatggatgaact atacaaaaggcctgctgcaaacgacgaaaactacgctttag tagcttaataa
RFP-lva (BBa_K411208)	Red fluorescence protein with degradation tag	atggcttcctccgaagacgttatcaaagagttcatgcgtttc aaagttcgtatggaaggttccgttaacggtcacgagttcga aatcgaaggtgaaggtgaaggtcgtccgtacgaaggtaccc agaccgctaaactgaaagttaccaaaggtggtccgctgccg ttcgcttgggacatcctgtccccgcagttccagtacggttcca aagcttacgttaaacacccggctgacatcccggactacctga aactgtccttcccggaaggtttcaaatgggaacgtgttatga acttcgaagacggtggtgttgttaccgttacccaggactcct ccctgcaagacggtgagttcatctacaaagttaaactgcgtg gtaccaacttcccgtccgacggtccggttatgcagaaaaaa accatgggttgggaagcttccaccgaacgtatgtacccgga agacggtgctctgaaaggtgaaatcaaaatgcgtctgaaac tgaaagacggtggtcactacgacgctgaagttaaaaccacc tacatggctaaaaaaccggttcagctgccgggtgcttacaa aaccgacatcaaactggacatcacctcccacaacgaagact acaccatcgttgaacagtacgaacgtgctgaaggtcgtcac tccaccggtgctgctgcaaacgacgaaaactacgctttagta gcttaataa

continues in the next page

Name	Description	Sequence
LacI-lva (BBa_C0012)	Inhibitor protein with degradation tag	atggtgaatgtgaaaccagtaacgttatacgatgtcgcaga<br>gtatgccggtgtctcttatcagaccgtttcccgcgtggtgaa<br>ccaggccagccacgtttctgcgaaaacgcgggaaaaagtgg<br>aagcggcgatggcggagctgaattacattcccaaccgcgtg<br>gcacaacaactggcgggcaaacagtcgttgctgattggcgt<br>tgccacctccagtctggccctgcacgcgccgtcgcaaattgt<br>cgcggcgattaaatctcgcgccgatcaactgggtgccagcg<br>tggtggtgtcgatggtagaacgaagcggcgtcgaagcctgt<br>aaagcggcggtgcacaatcttctcgcgcaacgcgtcagtgg<br>gctgatcattaactatccgctggatgaccaggatgccattgc<br>tgtggaagctgcctgcactaatgttccggcgttatttcttgat<br>gtctctgaccagacacccatcaacagtattattttctcccatg<br>aagacggtacgcgactgggcgtggagcatctggtcgcattg<br>ggtcaccagcaaatcgcgctgttagcgggcccattaagttct<br>gtctcggcgcgtctgcgtctggctggctggcataaatatctc<br>actcgcaatcaaattcagccgatagcggaacgggaaggcg<br>actggagtgccatgtccggttttcaacaaaccatgcaaatgc<br>tgaatgagggcatcgttcccactgcgatgctggttgccaacg<br>atcagatggcgctgggcgcaatgcgcgccattaccgagtcc<br>gggctgcgcgttggtgcggatatctcggtagtgggatacga<br>cgataccgaagacagctcatgttatatcccgccgttaaccac<br>catcaaacaggattttcgcctgctggggcaaaccagcgtgg<br>accgcttgctgcaactctctcagggccaggcggtgaagggc<br>aatcagctgttgcccgtctcactggtgaaaagaaaaaccac<br>cctggcgcccaatacgcaaaccgcctctccccgcgcgttggc<br>cgattcattaatgcagctggcacgacaggtttcccgactgga<br>aagcgggcaggctgcaaacgacgaaaactacgctttagtag<br>cttaataactctgatagtgctagtgtagatctc
pLac (BBa_K091112)	*LacI* repressible promoter	agcggcatgcatttacgttgacaccacctttcgcggtatggc<br>atgatagcgcccgg
pBAD (BBa_I13453)	Arabinose inducible promoter	acattgattatttgcacggcgtcacactttgctatgccatagc<br>atttttatccataagattagcggatcctacctgacgcttttta<br>tcgcaactctctactgtttctccataccgtttttttgggctagc
*RBS*34 (BBa_B0034)	Strong ribosome building site	aaagaggagaaa
*RBS*30 (BBa_B0030)	Weak ribosome building site	attaaagaggagaaa

TABLE II: List of DNA pieces used to build the synthetic SOC motif.

B. Plasmid Map

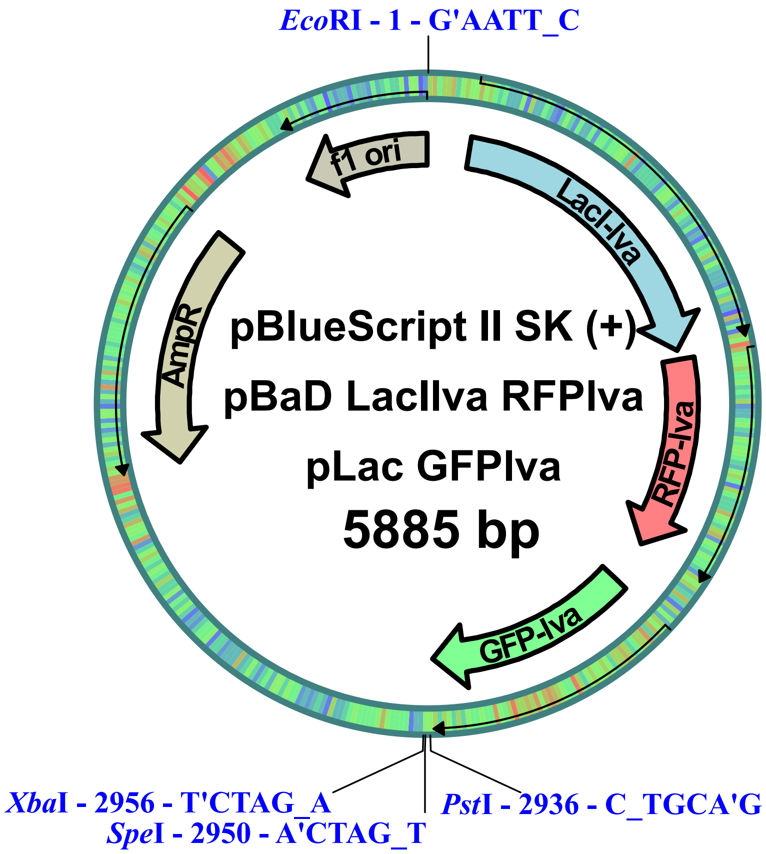

FIG. 12: Plasmid feature maps. The details of the plasmid construction can be found in the Section IV in the main manuscript. Expressed proteins are here depicted by arrows and the enzyme restriction sites from EcoRI, Pst,Spe, and XbaI are written in blue.

C. FACS analysis

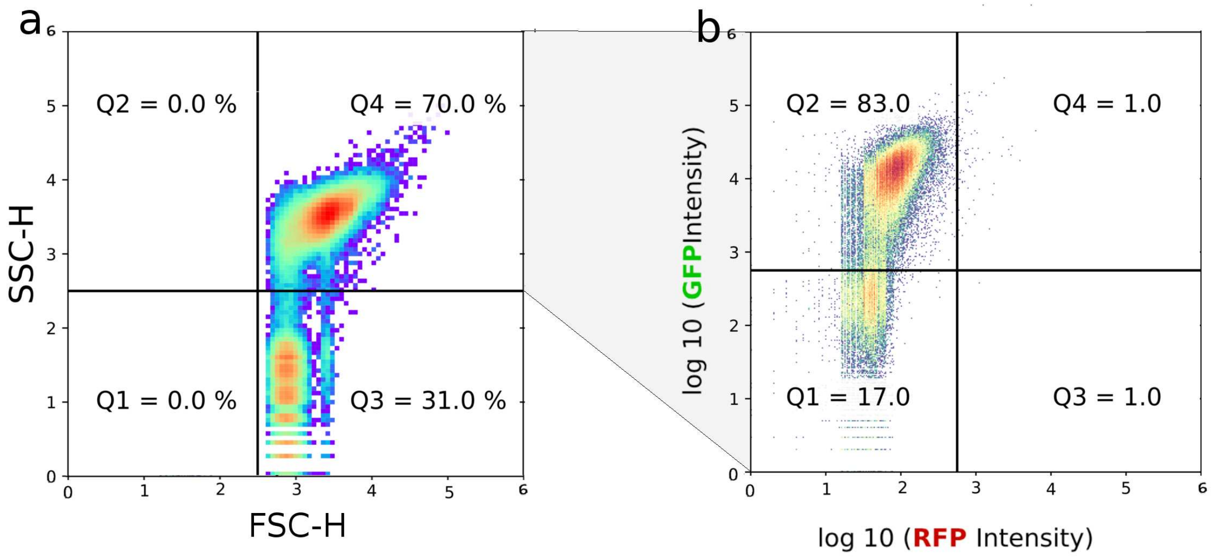

FIG. 13: FACS sequential gating strategy Flow cytometry dot plots of forward and side scattering channels (a) and specific emission fluorescence channels for GFP (FITC-H) and RFP (PE-H)(b).The gates of SSC-FSC panel (a) where positioned to avoid the debris. To facilitate this, we used an experimental condition where mostly all cells should exhibit GFP emission (with 10uM IPTG and 0 mM Arabinose). We read a total of 10^4 particles in Q4 for each sample of the experiments. Only the cells of Q4 where considered e.coli in healthy shape and their emission of fluorsecence was subsequently analyzed. The experiments were preformed as follows: one colony was grown in 4 ml LB at 37°C; this fresh exponential culture was used to inoculate fresh media. The culture was read in the FACS after 10h of incubation at 37°C.

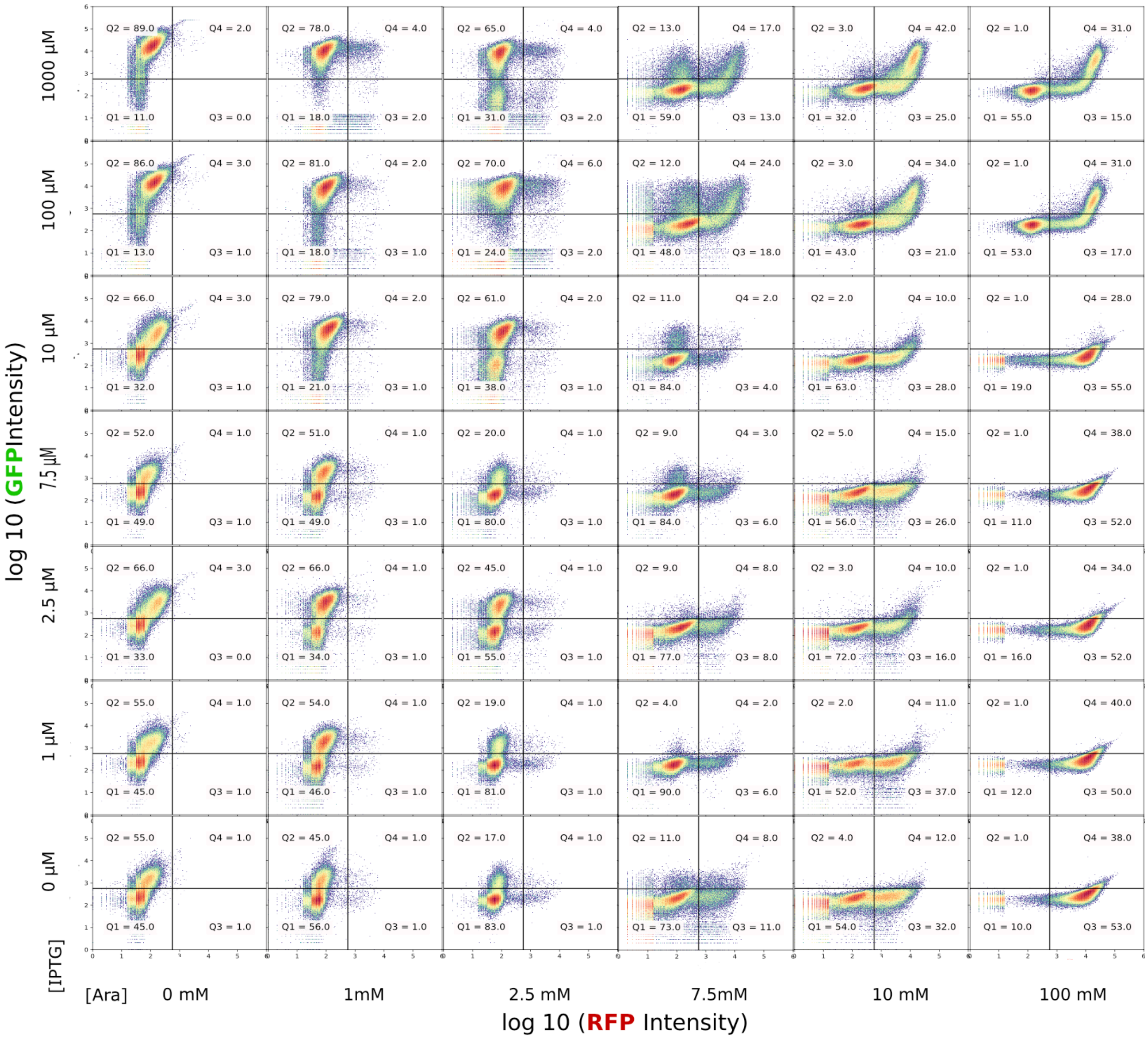

FIG. 14: Extended parameter space sweep, which allows to identify the conditions in which SOC occurs. Flow cytometry dot plots for different combinations of IPTG and Arabinose concentrations. One colony was grown in 4 ml LB at 37°C; this fresh exponential culture was used to homogeneously inoculate all the conditions of the experiment. After 10h of incubation at 37°C, a total of 10^4 cells were read from each sample. A proper gate to subtract the debris particles was set using forward and side scattering channels. Specific emission fluorescence channels for GFP (FITC-H) and RFP (PE-H) were plotted in adjacent axis.

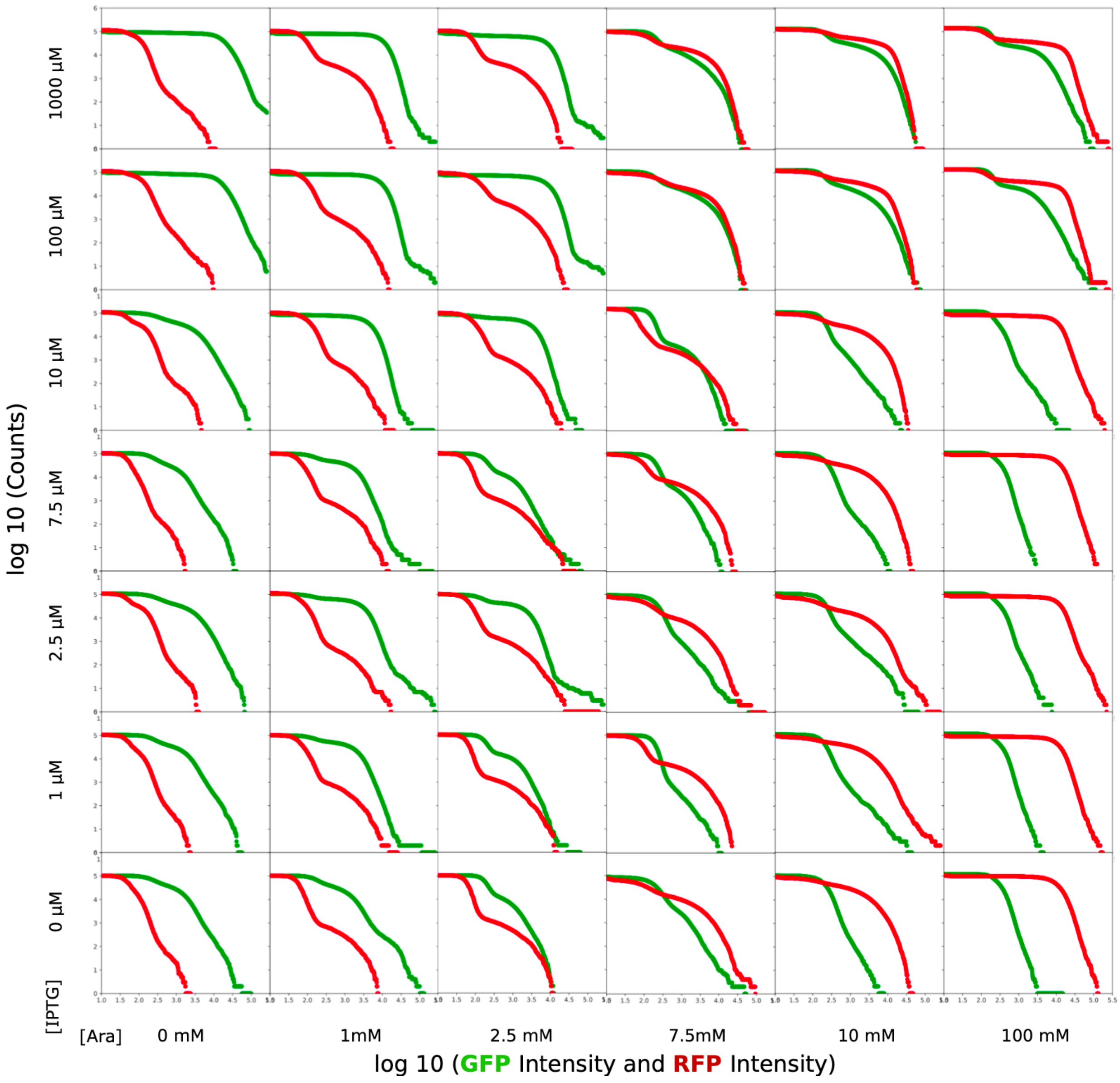

FIG. 15: Cumulative distributions of the experimental results shown in Fig. 14. Green and red lines depict the intensity of GFP and RFP fluorescence, respectively. SOC conditions are identified in the distributions with cumulative distributions of GFP exhibiting a straight line with a slope around 2 (all panels are plotted in log-log scale).

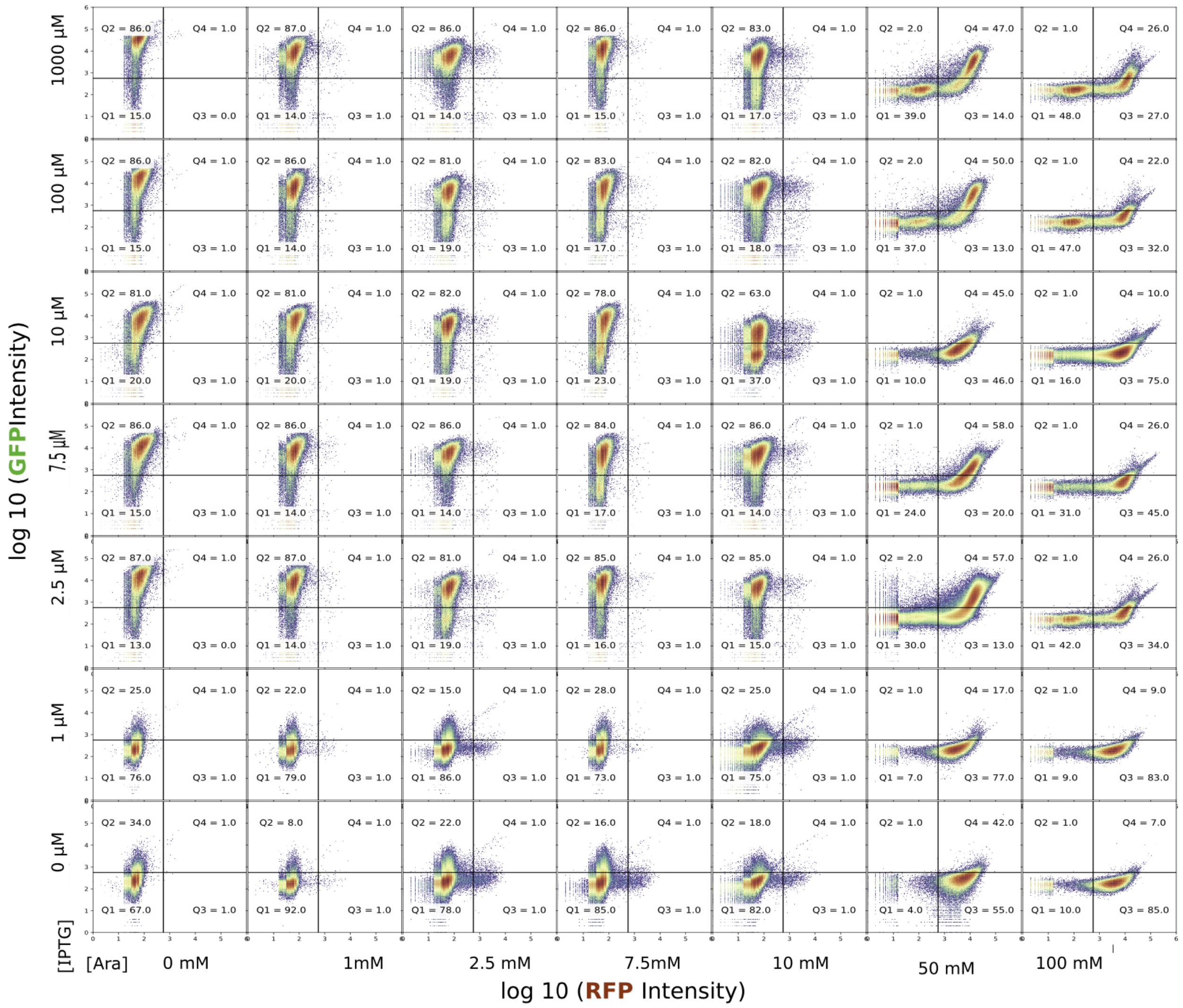

FIG. 16: Extended parameter space sweep, allowing to identify the conditions in which SOC occurs. Flow cytometry dot plots of different combinations of IPTG and Arabinose. One colony was grown in 4 ml LB at 37°C; this fresh exponential culture was used to homogeusly inoculate all the conditions of the experiment. After 10h of incubation at 37°C, a total of 10^4 cells were read from each sample. A proper gate to subtract the debris particles was set using forward and side scattering channels. Specific emission fluorescence channels for GFP (FITC-H) and RFP (PE-H) were plotted in adjacent axis.

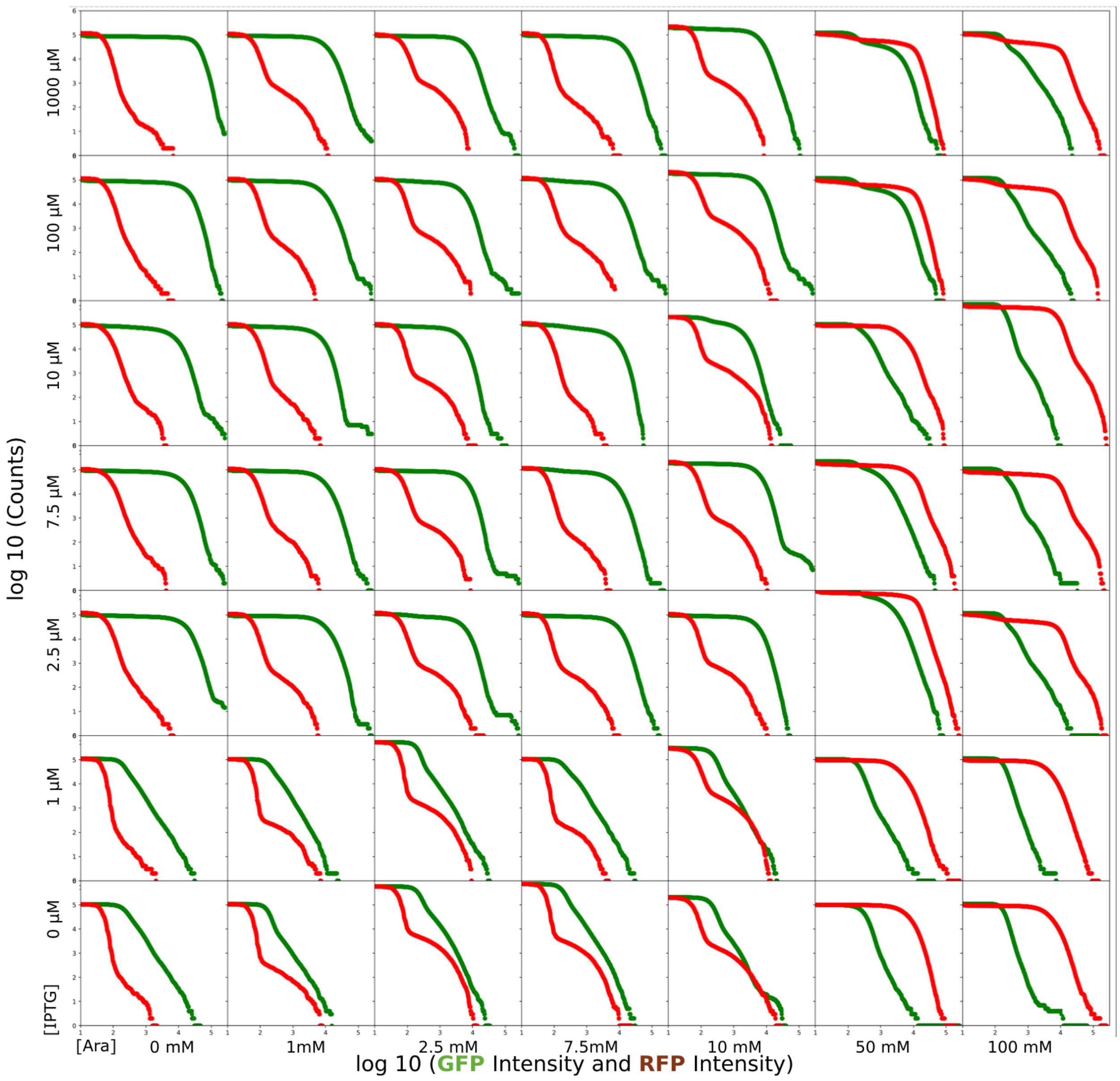

FIG. 17: Cumulative distributions of the above experiment. Green and red lines depict the intensity of GFP and RFP fluorescence, resepectively. SOC condition is identified in the conditions that allow the cumulative distribution of green to exhibit a straight line with a slope around 2.

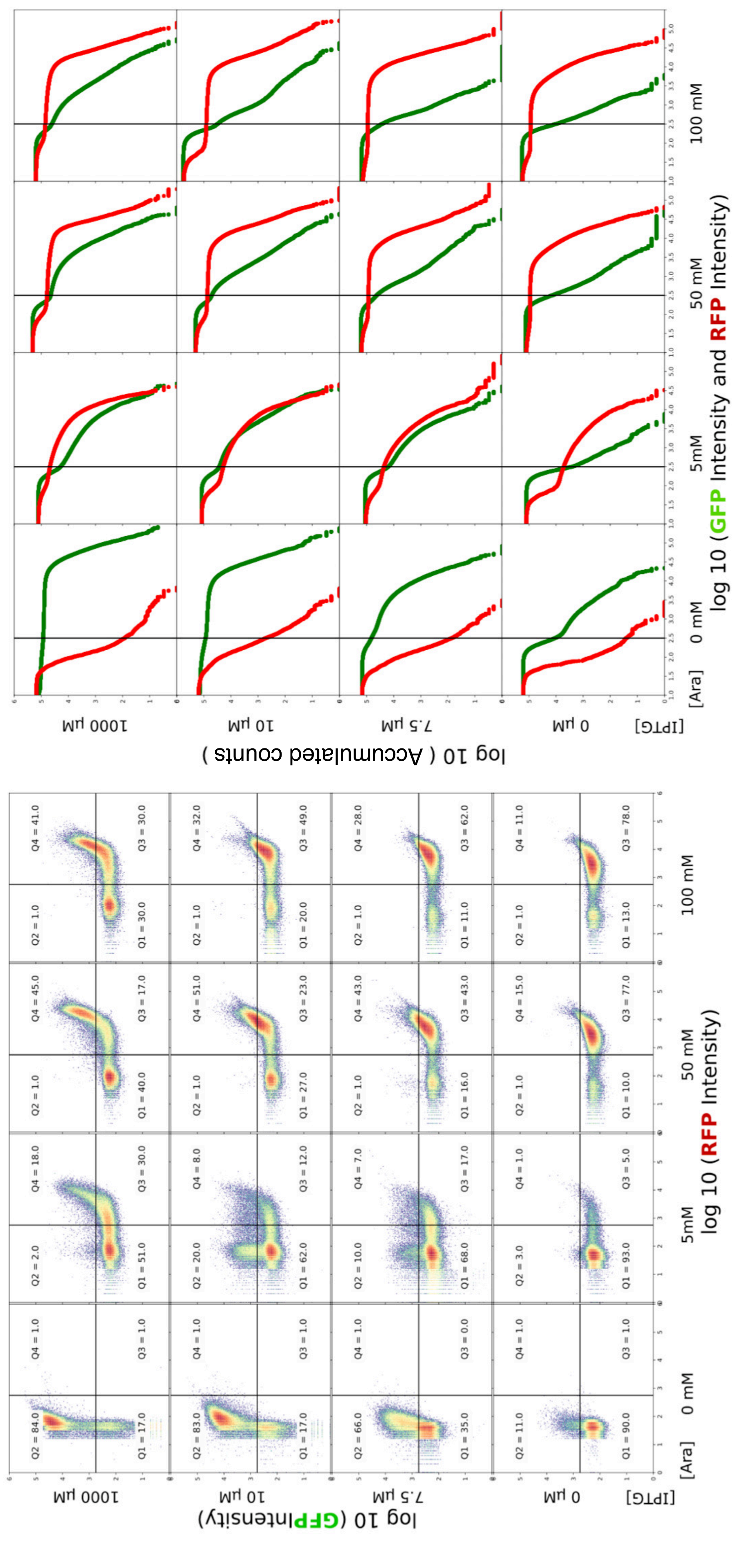

FIG. 18: FACS and cumulative distributions of one of three replicates of the experiment Flow cytometry dot plots of different combinations of IPTG and Arabinose. One colony was grown in 4 ml LB at 37°C; this fresh exponential culture was used to homogeneously inoculate all the conditions of the experiment. After 10h of incubation at 37°C, a total of 10^4 cells were read from each sample. A proper gate to subtract the debris particles was set using forward and side scattering channels. Specific emission fluorescence channels for GFP (FITC-H) and RFP (PE-H) were plotted in adjacent axis. Green and red lines depict the intensity of GFP and RFP fluorescence, respectively. SOC condition is identified in the conditions that allow the cumulative distribution of green to exhibit a straight line with a slope around 2.

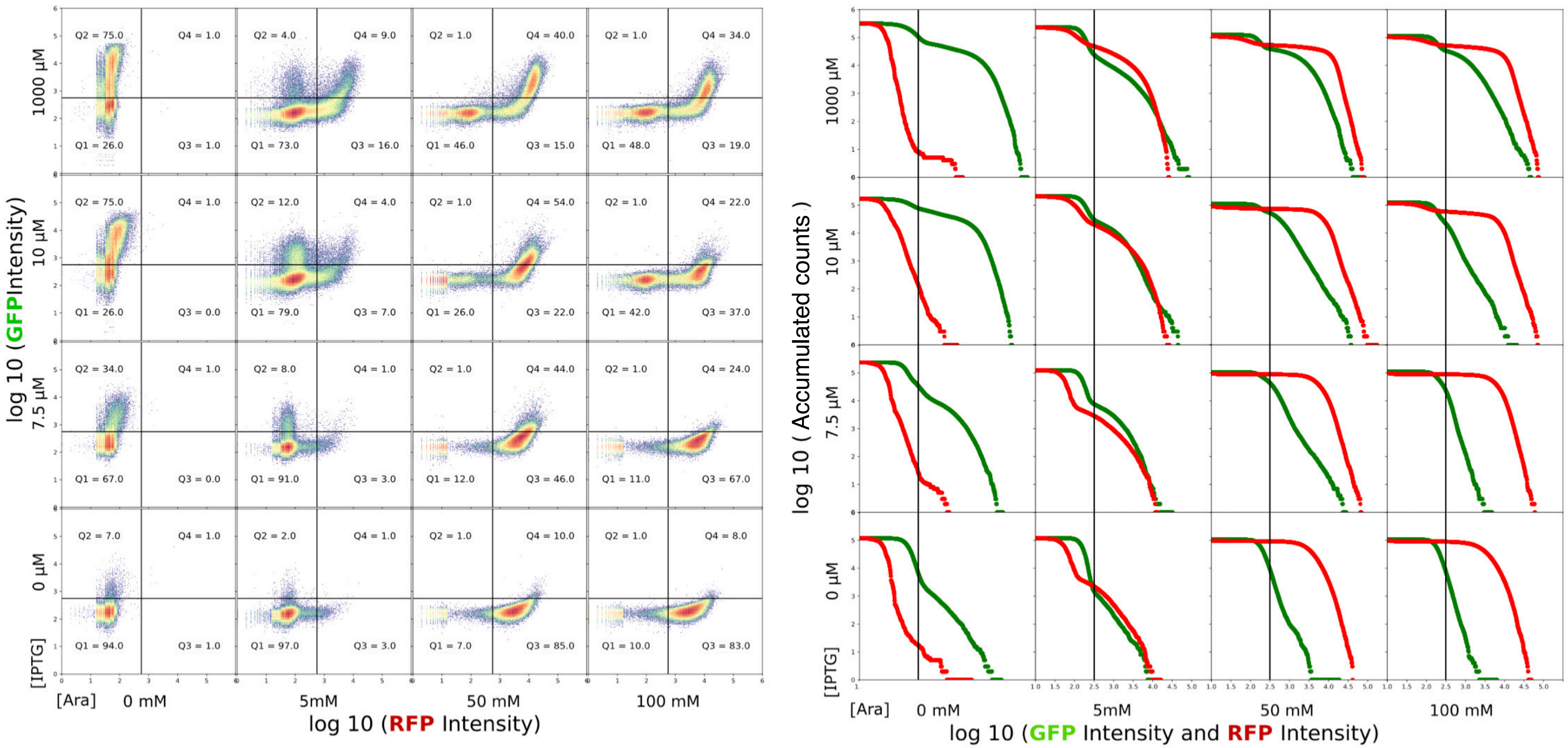

FIG. 19: FACS and cumulative distributions of two of three replicates of the experiment Flow cytometry dot plots of different combinations of IPTG and Arabinose. One colony was grown in 4 ml LB at 37°C; this fresh exponential culture was used to homogeneously inoculate all the conditions of the experiment. After 10h of incubation at 37°C, a total of 10^4 cells were read from each sample. A proper gate to subtract the debris particles was set using forward and side scattering channels. Specific emission fluorescence channels for GFP (FITC-H) and RFP (PE-H) were plotted in adjacent axis. Green and Red lines depict the intensity of GFP and RFP fluorescence, respectively. SOC condition is identified in the conditions that allow the cumulative distribution of green to exhibit a straight line with a slope around 2.

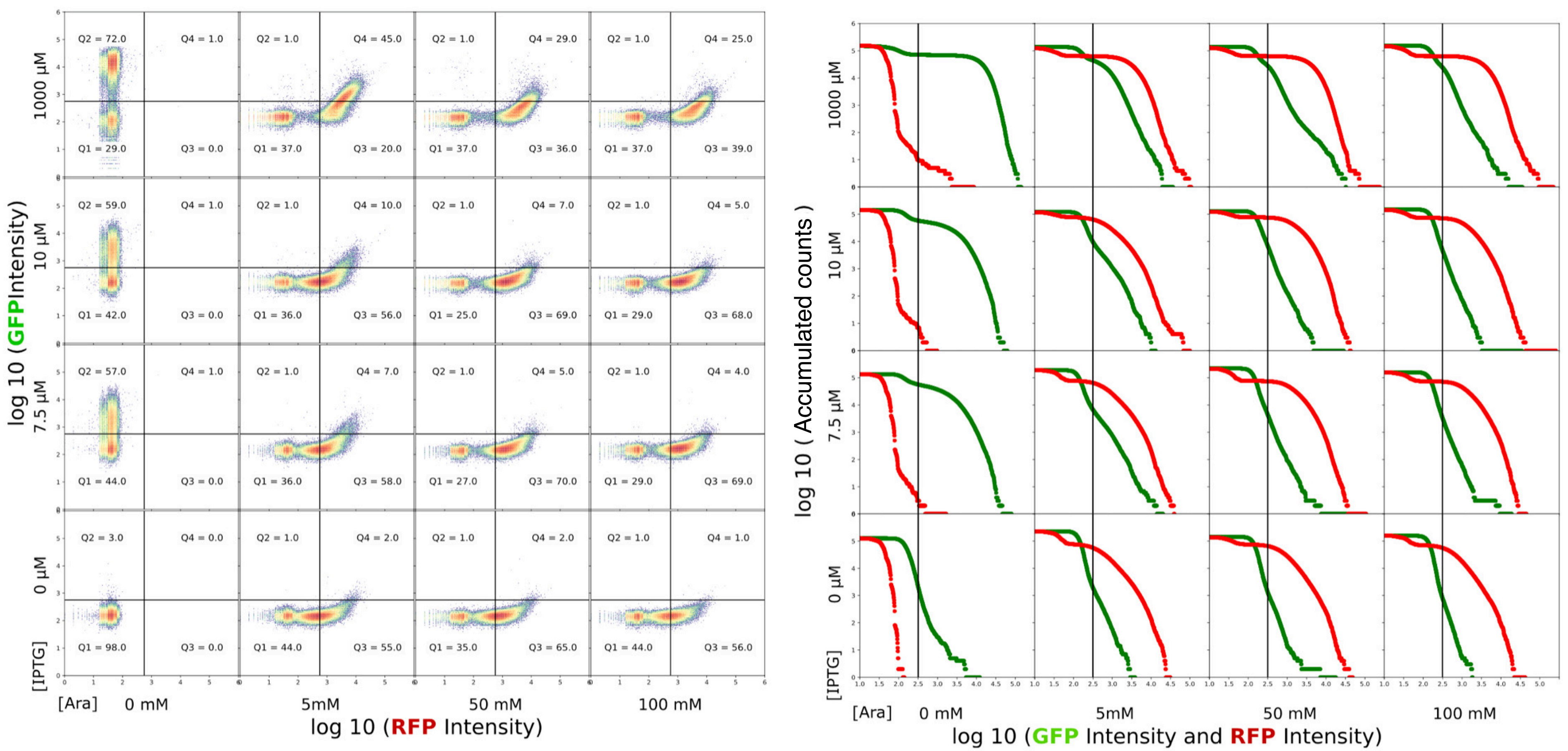

FIG. 20: FACS and cumulative distributions of three of three replicates of the experiment Flow cytometry dot plots of different combinations of IPTG and Arabinose. One colony was grown in 4 ml LB at 37°C; this fresh exponential culture was used to homogeneously inoculate all the conditions of the experiment. After 10h of incubation at 37°C, a total of 10^4 cells were read from each sample. A proper gate to subtract the debris particles was set using forward and side scattering channels. Specific emission fluorescence channels for GFP (FITC-H) and RFP (PE-H) were plotted in adjacent axis. Green and red lines depict the intensity of GFP and RFP fluorescence, respectively. SOC condition is identified in the conditions that allow the cumulative distribution of green to exhibit a straight line with a slope around 2.

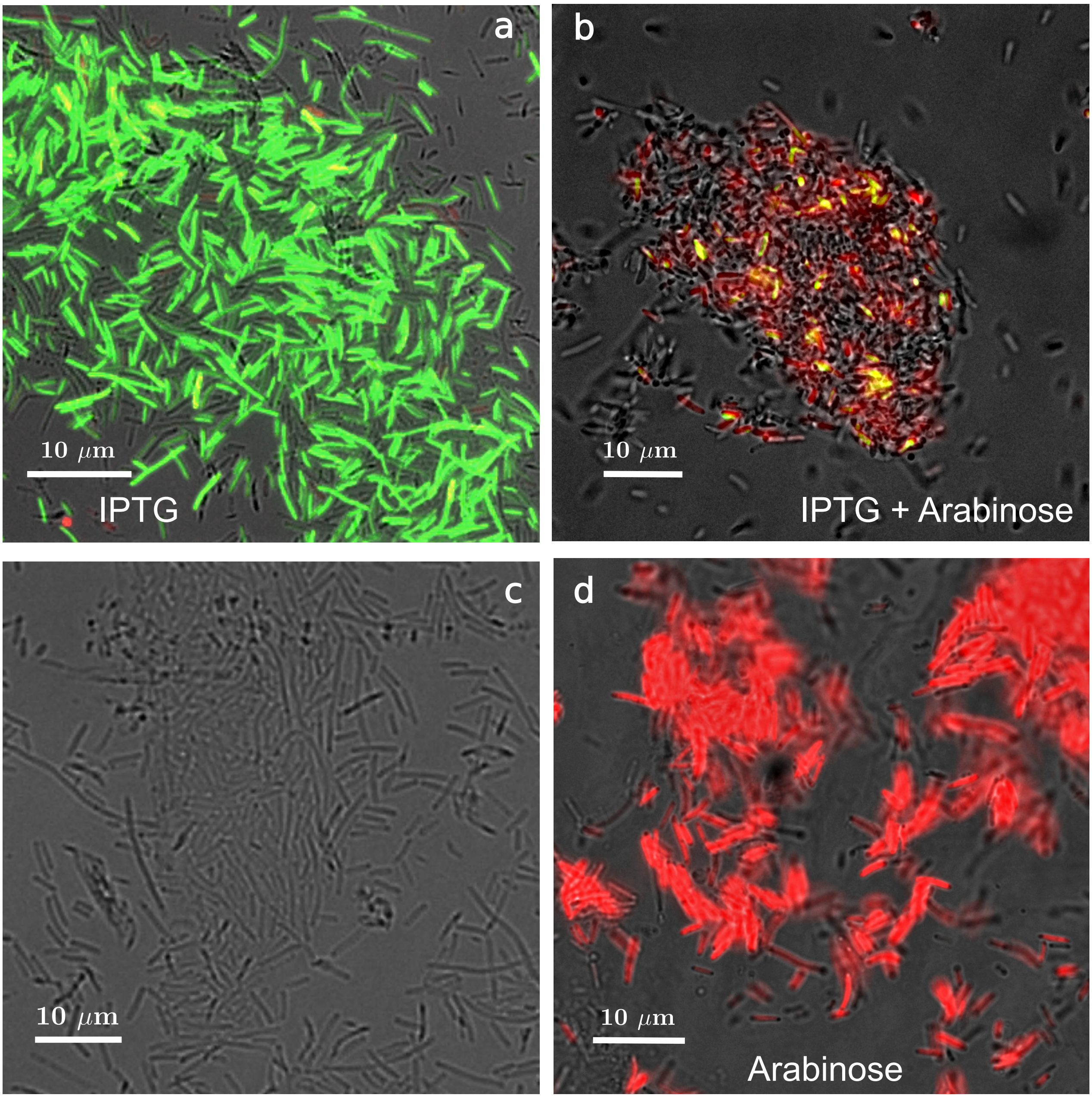

FIG. 21: Merge of brightfield and florescence microscope images with different combinations of IPTG and Arabinose (Ara). (c) No inputs. Without Ara, there is no production of LacI or the reporter protein RFP. In the absence of IPTG, endogenous LacI represses GFP production (very few bacteria with low-GFP intensity can be found). (d) The addition of 100 mM of Ara triggers LacI and RFP production. (a) In the absence of Ara but with 10μM IPTG, the inhibition of LacI triggers GFP expression. (b) Under the presence of 100 mM of Ara and 10μM IPTG, cells exhibit SOC behaviour. These time snapshots allow to observe qualitatively the combination of different possible expression profiles as we change parameters characteristic of SOC behaviour. These microscope images were taken using the bacteria cultures of the first replica of the main experiment, their FACS data is shown in figure 3 of the main text.

IV. REFERENCES

[1] Cookson, N.A., Mather, W.H., Danino, T., Mondragon, et al. 2011. Queueing up for enzymatic processing: correlated signaling through coupled degradation. *Mol. Syst. Biol.* 7(1): 561.

[2] Gillespie DT. 1976. A general method for numerically simulating the stochastic time evolution of coupled chemical reactions. *J. Comput. Phys.* 22: 403–434.

[3] Gillespie DT. 1977. Exact stochastic simulation of coupled chemical reactions. *J. Phys. Chem.* 81: 2340–2361.

[4] Gillespie DT. 2007 Stochastic Simulation of Chemical Kinetics. *Ann. Rev. Physical Chem.* 58(1): 35-55.

[5] Waskom M., Botvinnik O., Ostblom J. et al 2020 mwaskom/seaborn: v0.10.1 Zenodo.
