# Supplementary material for: Engineering self-organized criticality in living cells
Source: Nat Commun. 2021 Jul 20;12:4415. doi: 10.1038/s41467-021-24695-4 (PMC8292319; doi:10.1038/s41467-021-24695-4)
Supplement: Supplementary file 3 — Reporting Summary [file 41467_2021_24695_MOESM3_ESM.pdf]

nature research

Corresponding author(s): Ricard Sole and Nuria Conde

Last updated by author(s): June 02, 2021

Reporting Summary

Nature Research wishes to improve the reproducibility of the work that we publish. This form provides structure for consistency and transparency in reporting. For further information on Nature Research policies, see our Editorial Policies and the Editorial Policy Checklist.

Statistics

For all statistical analyses, confirm that the following items are present in the figure legend, table legend, main text, or Methods section.

n/a	Confirmed	
☐	☒	The exact sample size (n) for each experimental group/condition, given as a discrete number and unit of measurement
☐	☒	A statement on whether measurements were taken from distinct samples or whether the same sample was measured repeatedly
☒	☐	The statistical test(s) used AND whether they are one- or two-sided<br>*Only common tests should be described solely by name; describe more complex techniques in the Methods section.*
☒	☐	A description of all covariates tested
☐	☒	A description of any assumptions or corrections, such as tests of normality and adjustment for multiple comparisons
☒	☐	A full description of the statistical parameters including central tendency (e.g. means) or other basic estimates (e.g. regression coefficient) AND variation (e.g. standard deviation) or associated estimates of uncertainty (e.g. confidence intervals)
☒	☐	For null hypothesis testing, the test statistic (e.g. F, t, r) with confidence intervals, effect sizes, degrees of freedom and P value noted<br>*Give P values as exact values whenever suitable.*
☒	☐	For Bayesian analysis, information on the choice of priors and Markov chain Monte Carlo settings
☒	☐	For hierarchical and complex designs, identification of the appropriate level for tests and full reporting of outcomes
☒	☐	Estimates of effect sizes (e.g. Cohen's d, Pearson's r), indicating how they were calculated

Our web collection on statistics for biologists contains articles on many of the points above.

Software and code

Policy information about availability of computer code

Data collection	Flow Jo V7, Image J 1.8.0_172, Facs Diva software 9, Leica LAS X v3.3
Data analysis	Numpy 1.20.3, Matplotlib 3.4.2 , Scipy 1.6.3, FlowCytometryTools 0.5.1, powerlaw-1.4.6, Pandas 1.2.4, DiffEqJump v6.14.1, Catalyst v6.12.1

For manuscripts utilizing custom algorithms or software that are central to the research but not yet described in published literature, software must be made available to editors and reviewers. We strongly encourage code deposition in a community repository (e.g. GitHub). See the Nature Research guidelines for submitting code & software for further information.

Data

Policy information about availability of data

All manuscripts must include a data availability statement. This statement should provide the following information, where applicable:

- Accession codes, unique identifiers, or web links for publicly available datasets
- A list of figures that have associated raw data
- A description of any restrictions on data availability

The data that support the findings of this study are available from the corresponding authors upon reasonable request.Nonetheless, the source data is available in the open source repository OFS https://osf.io/h5cew/?view_only=33c12a4780954fcdbd9f1c0986adfdc6

Field-specific reporting

Please select the one below that is the best fit for your research. If you are not sure, read the appropriate sections before making your selection.

☒ Life sciences ☐ Behavioural & social sciences ☐ Ecological, evolutionary & environmental sciences

For a reference copy of the document with all sections, see nature.com/documents/nr-reporting-summary-flat.pdf

Life sciences study design

All studies must disclose on these points even when the disclosure is negative.

Sample size	10^4 bacteria were analyzed in the FACS Cytometer for each experiment for each condition. The 10^4 number of cells (events inside the devris-free cell gate) was already been proven enough to previous synthetic biocomputation research of the group (https://doi.org/10.1371/journal.pcbi.1004685), and is within the recomended amount of events to be recordet when reading bacteria with FACS (https://doi.org/10.1371/journal.pone.0240233). We also fast-check that increasing the lecture to 10^5 does not alter the qualitative result or the quantitative percentages of the different behaviours of the circuit (data not shown).
Data exclusions	There has not been data exclusion (only the filtering of particles in the flow cytometer). See replication statement
Replication	This is not relevant for our study. The bacterial samples were composed of only one strain bacteria and exposed to different conditions to perform the experiment. Each experiment consist in 4 ml culture at OD=0.8 which contains around 4x10^8 bacterial cells.The flow cytometer machine randomly reads 10^4 of them. For the same specified growth conditions, the experiments were successful to replicate the results. Each experiment was replicated 3 independent times with same growth conditions
Randomization	This is not relevant for our study. The bacterial samples were composed of only one strain of bacteria and exposed to different conditions to perform the experiment.
Blinding	Blinding does not apply to our expermients

Reporting for specific materials, systems and methods

We require information from authors about some types of materials, experimental systems and methods used in many studies. Here, indicate whether each material, system or method listed is relevant to your study. If you are not sure if a list item applies to your research, read the appropriate section before selecting a response.

Materials & experimental systems

n/a	Involved in the study	
☒	☐	Antibodies
☒	☐	Eukaryotic cell lines
☒	☐	Palaeontology and archaeology
☒	☐	Animals and other organisms
☒	☐	Human research participants
☒	☐	Clinical data
☒	☐	Dual use research of concern

Methods

n/a	Involved in the study	
☒	☐	ChIP-seq
☐	☒	Flow cytometry
☒	☐	MRI-based neuroimaging

Flow Cytometry

Plots

Confirm that:

☒ The axis labels state the marker and fluorochrome used (e.g. CD4-FITC).

☒ The axis scales are clearly visible. Include numbers along axes only for bottom left plot of group (a 'group' is an analysis of identical markers).

☒ All plots are contour plots with outliers or pseudocolor plots.

☒ A numerical value for number of cells or percentage (with statistics) is provided.

Methodology

Sample preparation	Single colonies of our XL1 e.coli freshly transformed with our plasmid were inoculated in 4 ml of LB media supplemented with ampicillin and grown at 37ºC with shaking (200 r.p.m.) during 4 hours, to reach an approximate OD660 of 0.6. One microliter of the culture was re-inoculated in 4ml of fresh LB media, supplemented with ampicillin, and the corresponding input

	concentrations. The cultures were grown during 10 hours at 37ºC with shaking. Once they were at OD660 of 0.8-1, were used for fuorescence measures.
Instrument	BD LSRFortessa (lasers: 405 - 488 - 561 - 633, detectors:FSC/SSC + 14 PMT)<br>Leica DMI6000 Inverted
Software	Diva 8, FlowJo V7, FlowCytometryTools 0.5.1, powerlaw-1.4.6
Cell population abundance	As already mentioned a total of 10^4 were analyzed from each sample. All bacteria cultures were constituted by only one type of bacterial strain, and the population percentages (the different levels of fluorescence exhibited) are the result of the circuit behaviour (the result of the experiment). Which are explained in detail in the article.
Gating strategy	A proper gate to subtract the debris particles was set using forward and side scattering channels. This gate was determined once using a bacterial population of 100% florescent bacteria. The cumulative distributions depict all bacteria with a FITC-H expression above 10^2.5.

☒ Tick this box to confirm that a figure exemplifying the gating strategy is provided in the Supplementary Information.
